# Exploring the molecular mechanism of dexmedetomidine in alleviating blood–brain barrier disruption in rats with cerebral ischemia reperfusion injury based on network pharmacology

**DOI:** 10.3389/fnmol.2026.1750882

**Published:** 2026-04-08

**Authors:** Xue Lv, Wei Gao, Zhi-Guo Zhang, Jian-Xin Jia

**Affiliations:** 1 Department of Human Anatomy, Baotou Medical College, Baotou, China; 2 Department of Anesthesiology, The Third Hospital of Baogang Group, Baotou, China; 3 Key Laboratory of Human Anatomy, Education Department of Inner Mongolia Autonomous Region, Baotou, China

**Keywords:** AMPK/mTOR signaling pathway, autophagy, blood–brain barrier, cerebral ischemia reperfusion injury, dexmedetomidine, network pharmacology

## Abstract

**Objective:**

This study aimed to clarify the neuroprotective effect of dexmedetomidine (DEX) against cerebral ischemia reperfusion injury (CIRI) and its underlying mechanism using network pharmacology and *in vivo* validation.

**Methods:**

Network pharmacology was employed to explore the mechanism underlying DEX-mediated alleviation of CIRI. A rat CIRI model was established using the suture-occlusion method. Neurological scoring and behavioral assessments were conducted to evaluate neurological and motor functions; histological examination was performed to observe brain tissue and blood–brain barrier (BBB) injury. Western blotting and immunofluorescence analysis were utilized to assess the protein levels of factors associated with BBB integrity.

**Results:**

Network pharmacology analysis revealed that DEX may exert a protective effect against CIRI through the AMPK/mTOR signaling pathway. DEX treatment significantly attenuated CIRI-induced impairments in neurological function and motor performance. Specifically, DEX upregulated the protein expression levels of P-AMPK/AMPK ratio, beclin 1, LC3 II/I, and ZO-1, whereas the P-mTOR/mTOR ratio and P62 were significantly downregulated, and cerebral tissue injury was alleviated.

**Conclusion:**

DEX exerts a significant protective effect against BBB injury in rats with CIRI. This neuroprotective effect is mediated by multiple synergistic mechanisms, including the upregulation of tight junction proteins and the regulation of the AMPK/mTOR signaling pathway. Collectively, the findings of the present study suggest that DEX represents a promising potential agent for the clinical treatment of CIRI-associated BBB impairment.

## 1 Introduction

Ischemic stroke is the predominant subtype of stroke, accounting for approximately 85% of all cases, with the remainder being hemorrhagic stroke (Luo et al., 2025). Clinically, the primary therapeutic strategies for ischemic stroke include mechanical thrombectomy and thrombolysis with recombinant tissue plasminogen activator (rtPA), both designed to promptly restore blood perfusion to the affected cerebral tissue (Zhao et al., 2025). However, reperfusion of the previously ischemic region can trigger secondary pathological cascades such as inflammation and necrosis, a phenomenon termed cerebral ischemia reperfusion injury (CIRI) (Xu et al., 2025). CIRI is associated with severe neurological deficits, including cognitive impairment, motor dysfunction, and depressive symptoms. The underlying pathophysiology of CIRI involves multiple mechanisms, including blood–brain barrier (BBB) disruption, inflammatory responses, and cellular apoptosis (Bao et al., 2025). Among these, BBB damage plays a pivotal role in exacerbating the progression and severity of CIRI.

The central nervous system (CNS) maintains a highly specialized and dynamically stable intracerebral microenvironment, which is indispensable for normal neural function. As a critical interface between the bloodstream and neural tissue, the BBB primarily governs bidirectional substance transport, ensures the delivery of essential nutrients, and plays a pivotal role in preserving cerebral homeostasis (Zhang Q. et al., 2025). Structurally, the BBB comprises cerebral microvascular endothelial cells, endothelial tight junction proteins (TJs), neuroglia, astrocytes, pericytes, and the basement membrane (Dai et al., 2025). Among these components, TJs serve as the structural cornerstone of the BBB, regulating paracellular and transmembrane transport of various substances. These TJs are predominantly composed of transmembrane and adhesion proteins, including occludin, claudin-5 and zonula occludens-1 (ZO-1), as well as cytoskeletal proteins (Berselli et al., 2025). Notably, ZO-1 and claudin-5 are the most abundant and are localized at the lateral borders of endothelial cells. They function to anchor adjacent cells, seal intercellular clefts, and prevent the passage of extracellular macromolecules into the tissue parenchyma through these gaps (Lee et al., 2025). During the pathophysiological process of CIRI, TJs associated with the BBB undergo irreversible structural damage, accompanied by abnormal cytoskeletal remodeling and disorganization in cerebral microvascular endothelial cells (Song et al., 2023). These pathological alterations lead to a marked increase in BBB permeability or even complete barrier breakdown, permitting the extravasation of intravascular macromolecules and the influx of exogenous harmful substances into the CNS (Zhou et al., 2021). This cascade of events further exacerbates primary and secondary brain tissue damage. Importantly, the structural integrity and functional expression of TJs directly govern the permeability of the BBB.

Dexmedetomidine (DEX), a highly selective α2-adrenoceptor agonist with sedative, analgesic, and opioid-sparing properties, is widely utilized in the management of critically ill patients and in clinical anesthetic practice (Zhou et al., 2025). Beyond its well-documented sedative and analgesic effects, accumulating evidence has demonstrated that DEX possesses anti-inflammatory, anti-oxidative stress, and anti-apoptotic activities, while exerting protective effects on ischemic organs and the nervous system (Li et al., 2022; Unchiti et al., 2021). Recent studies have further revealed that DEX promotes angiogenesis in murine models of ischemic stroke and mitigates vascular endothelial cell injury via activation of the PI3K/Akt signaling pathway (Kim et al., 2025). Additionally, it has been shown to facilitate neuronal regeneration through the activation of the BDNF/ TrkB/CREB signaling pathway (Saral et al., 2024). Furthermore, mounting evidence indicates that DEX ameliorates intestinal barrier dysfunction following intestinal ischemia (Feng T. et al., 2025; Gu et al., 2025). However, the specific mechanism by which DEX alleviates BBB damage in rats with CIRI remains largely unelucidated. To address this critical knowledge gap, the present study employs high-throughput sequencing technology to explore the potential molecular pathways underlying DEX mediated BBB protection.

With the rapid advancement of bioinformatics, network pharmacology has evolved into a robust tool for investigating complex pharmaceutical compounds and their biological functions. It is widely employed to identify potential therapeutic components in both traditional Chinese and Western medicines, while also predicting their underlying pharmacological targets at the molecular level. First, we hypothesized that DEX may mitigate BBB damage induced by CIRI through the adenosine monophosphate-activated protein kinase (AMPK)/mammalian rapamycin target protein (mTOR) signaling pathway. Subsequent experiments were designed to validate this proposed mechanism. Utilizing middle cerebral artery occlusion (MCAO) model rats, we investigated whether DEX treatment could alleviate CIRI severity by upregulating the expression of TJs, thereby enhancing BBB integrity. Compound C, also known as dorsomorphin, is a synthetic small molecule compound belonging to pyrazolopyrimidine derivatives. It is widely used as a selective, ATP-competitive inhibitor of AMPK. In this study, we used this compound to further explore the specific mechanism of the AMPK/mTOR signaling pathway. By capitalizing on the clinical accessibility of DEX, a pharmacologic agent routinely administered preoperatively, this study offers deeper and more comprehensive insights into DEX as a promising therapeutic candidate for CIRI.

## 2 Materials and methods

### 2.1 Animals

Adult male Wistar rats (260–280 g) were obtained from Beijing SpePharm Biotechnology Co., Ltd. All animals were housed in the Animal Experiment Center of Baotou Medical College, Inner Mongolia University of Science and Technology, with an ambient temperature of 23 ± 1 °C, a humidity of 50%, and a 12 h light/dark cycle, with ad libitum access to food and water. The experimental protocol was approved by the Animal Ethics Review Committee of Baotou Medical College [Approval No. (2022) 60]. Animals were randomly assigned to four experimental groups (*n* = 6 per group): (a) Sham group: underwent identical surgical procedures without insertion of the suture; (b) MCAO group: subjected to MCAO for 2 h followed by 24 h of reperfusion; (c) DEX group: received an intraperitoneal injection of DEX (50 μg/kg) 30 min prior to MCAO, followed by 2 h of ischemia and 24 h of reperfusion; (d) Compound C + DEX (C + D) group: treated with DEX (50 μg/kg) 30 min prior to MCAO and Compound C (a specific small-molecule AMPK antagonist; 20 mg/kg; intraperitoneal injection) 25 min prior to MCAO, followed by 24 h of reperfusion. All procedures were conducted in accordance with the university guidelines for animal care and use and complied with the ARRIVE (Animal Research: Reporting of *In Vivo* Experiments) guidelines. All rats in the behavioral tests were included in the analyses except for those with severe skin wounds or more than 15% body weight loss. Both the treatments and the data analyses were blinded to the experimenters. All experiments in this study were performed using tissues from the ischemic penumbra to improve the accuracy of the research.

### 2.2 Network pharmacology

#### 2.2.1 Acquisition and screening of components and targets

The components and targets of DEX were retrieved and collected through the PubChem database and literature mining. Subsequently, the active components of DEX and their Simplified Molecular-Input Line-Entry System (SMILES) codes were obtained and imported into the SwissTargetPrediction database for target prediction. The keyword “CIRI” was searched and screened in the GeneCard database and Online Mendelian Inheritance in Man (OMIM) database to acquire relevant targets. The Venny web tool was used to identify the common targets of DEX and CIRI.

#### 2.2.2 Construction of the “component-target-disease” visual network

Common targets of CIRI and DEX were imported into Cytoscape 3.9.2 software for network topological analysis, and a visual “component-target-disease” network was constructed.

#### 2.2.3 Construction of protein–protein interaction network

The common targets of CIRI and DEX were imported into the STRING database to construct a protein–protein interaction (PPI) network. *Homo sapiens* was set as the restricted organism, and the required minimum interaction score was set to the highest confidence level (0.900). Finally, Cytoscape 3.9.2 software was used to map the PPI network, with the top ten-degree values as the core indicator.

#### 2.2.4 GO enrichment analysis

The common targets of CIRI and DEX were imported into ClusterProfiler and Stringin software for GO (Gene Ontology) enrichment analysis (*p* ≤ 0.01) to clarify the target functions of DEX’s active components. Subsequently, the top 10 targets were selected individually and visualized using a bioinformatics platform.

#### 2.2.5 KEGG analysis

ClusterProfiler and Stringin software were used to perform KEGG (Kyoto Encyclopedia of Genes and Genomes) pathway analysis on key targets, aiming to explore the specific pathways through which DEX affects CIRI. The top 10 pathways with a cutoff value of *p* < 0.05 were retained.

### 2.3 Drugs and reagents

Compound C was obtained from MedMol (China), batch number S81448. DEX was purchased from Yang Zi Jiang Group Pharmaceuticals Co., Ltd. (China). All reagents were stored and prepared according to the manufacturers’ instructions.

### 2.4 MCAO rat model

The MCAO model was established using a modified Zea-Longa intraluminal suture technique (Longa et al., 1989). Rats were anesthetized with 2% sodium pentobarbital prior to surgery. The left common carotid artery (CCA), external carotid artery (ECA), and internal carotid artery (ICA) were carefully isolated and exposed. Following ligation of the distal CCA, a small incision was made to allow insertion of a nylon suture (length: approximately 45 mm, tip diameter: 0.36 ± 0.02 mm) into the ICA. To induce cerebral ischemia, the suture was advanced through the ICA until it reached and occluded the middle cerebral artery (MCA), targeting the intracranial segment of the ICA. After 2 h of occlusion, the suture was carefully withdrawn to the junction of the ECA and ICA to initiate a 24 h reperfusion period.

### 2.5 Neurological function assessment using the Zea-Longa scoring system

Neurological function was evaluated 24 h after reperfusion using the Zea-Longa scoring system. The evaluation criteria were defined as follows: 0 point: no neurological deficit and normal motor performance; 1 point: inability to fully extend the contralateral forepaw; 2 points: circling behavior toward the contralateral side during crawling; 3 points: falling to the contralateral side while walking; 4 points: inability to walk independently accompanied by loss of consciousness (Longa et al., 1989).

### 2.6 Open field test

To evaluate spontaneous locomotor activity, rats from each experimental group were placed individually in an open field test box. The travel distance was recorded for 5 min. Each test was repeated three times, and the results were expressed as the average value from six animals per group (Wang et al., 2022).

### 2.7 Sticker removal test

A rectangular sticker (1.2 × 2.0 cm) was affixed to the plantar surface of the forelimb of each rat. All animals underwent preoperative sticker removal training for three consecutive days prior to surgery, with two training sessions per day. Only rats that successfully removed the sticker within 30 s during training were included in the study. At 24 h post-operation, the time required for each rat to remove the sticker was recorded (Povinelli et al., 1996).

### 2.8 Balance bar test

A custom-built balance bar (150 cm × 4 cm × 3 cm; length × width × height) was supported by wooden frames at both ends, elevating it 80 cm above the ground. One end of the bar was defined as the starting point and the other as the endpoint. A rat cage was placed immediately behind the endpoint to serve as an incentive, while a 1 m × 1 m × 12 cm shock absorbing cushion was positioned beneath the beam to prevent injury in case of falls. Prior to formal testing, all rats underwent adaptive training for three consecutive days (three sessions/day) to familiarize themselves with the apparatus. For each test, a rat was gently placed at the starting point, facing the rat cage. The time required for the rat to traverse the entire bar and reach the endpoint was recorded. Baseline crossing times were measured preoperatively, and post-operative measurements were obtained separately for each group following MCAO. A maximum cutoff time of 120 s was set for bar crossing, with any rat failing to complete the task within this period assigned a score of 120 s (Pertwee and Greentree, 1988).

### 2.9 Nissl staining

Nissl staining was conducted as previously described (Fuyun et al., 2025). Paraffin-embedded brain sections were stained with 0.5% cresyl violet solution at room temperature for 10 min, followed by a brief immersion in 0.25% glacial acetic acid for several seconds. The sections were dehydrated through a graded ethanol series and cleared in xylene. The stained sections were then visualized under a light microscope for image acquisition (Lv et al., 2025).

### 2.10 Hematoxylin and eosin staining

Hematoxylin and eosin (HE) staining was conducted as previously described (Samur et al., 2025), strictly following the manufacturer’s protocol for the HE staining kit (Beijing Solarbio, China). After staining, brain sections were dehydrated via a graded ethanol series and mounted with neutral balsam (Lv et al., 2025).

### 2.11 2,3,5-Triphenyltetrazolium chloride staining

2,3,5-Triphenyltetrazolium chloride (TTC) staining was employed to quantify the infarct volume. At 24 h postoperatively, animals were anesthetized with 2% sodium pentobarbital, and whole brains were harvested. Brains were frozen at −20 °C for 30 min and subsequently sectioned into five coronal slices. These slices were stained with 2% TTC solution at 37 °C for 30 min in the dark. Stained brain tissues were placed on a plate for photography. Animals exhibiting blood clots or arterial thrombosis were excluded from the study. Infarct areas were quantified using Image-Pro Plus 6.0 software, and the percentage of infarct volume was calculated relative to the total brain area (Lou et al., 2025).

### 2.12 Evans blue assay

After MCAO, rats received an intravenous injection of 2% Evans blue solution. Two hours after the Evans blue injection, animals were anesthetized, and brains were rapidly removed. Brain tissues were homogenized in acetone, and the resulting supernatants were collected by centrifugation (Zhang S. et al., 2025). Absorbance of Evans blue was measured at 620 nm using an 800 TS microplate reader (BioTek, United States).

### 2.13 Transmission electron microscopy

The excised brain tissue samples were thoroughly fixed with glutaraldehyde, rinsed with phosphate buffered saline (PBS), and then fixed with osmic acid. Subsequently, the samples were embedded in resin and cut into ultra-thin sections with a thickness of 0.06 μm, which were then observed under a transmission electron microscope. Under transmission electron microscopy (TEM), the ultrastructure of the rat cortical tissue was examined, with specific focus on structures such as TJs and autophagosomes. This allowed for the evaluation of the structure and ultrastructural morphology of the BBB (Pelyuntha et al., 2025).

### 2.14 Immunofluorescence staining

Brain sections were incubated in 5% bovine serum albumin (BSA) for 1 h at room temperature to block non-specific binding, followed by overnight incubation at 4 °C with the primary antibodies (1:200 dilution). After three washes with PBST (0.1% Triton X-100 in 0.1 M PBS), sections were incubated with a fluorophore conjugated secondary antibodies (1:500 dilution) for 1 h at room temperature in the dark. Nuclei were counterstained with DAPI for 5 min. Fluorescent images were acquired using an ultra-high resolution inverted confocal microscope (Stellaris 5, Leica) equipped with appropriate excitation/emission filters. Z-stack images were captured at 20× magnification with a 0.5 μm step size. Vascular length and tip cell counts were quantified using ImageJ software (NIH, United States) with the Angiogenesis Analyzer plugin. All image analysis was performed blinded to experimental conditions to ensure objectivity (Shen et al., 2025).

### 2.15 Western blot

Following anesthesia and sacrifice, rat brains were removed and harvested. The cerebral cortex was isolated, and tissues were homogenized in RIPA buffer (Beyotime, Nantong, Jiangsu, China), with a grinder for 1 min, and then frozen at −20 °C for 30 min. The tissue lysate was centrifuged at 12,000 rpm at 4 °C for 15 min, and the supernatant was collected. Protein concentrations were determined using a BCA protein assay kit (Thermo Fisher Scientific, United States). Protein samples were mixed with SDS-PAGE protein loading buffer at a 4:1 volume ratio, and denatured in a metal bath at 100 °C for 10 min.

Electrophoresis and blotting were performed following the procedures described previously (Wen et al., 2004). The following rabbit-derived primary antibodies were used: AMPK, P-AMPK, Mtor, P-mTOR and ZO-1 (Wuhan Sanying Biotechnology Co., Ltd.), Goat anti-rabbit secondary antibodies (Shanghai Sabo Biotechnology Co., Ltd.) were used for detection (Shen et al., 2025).

### 2.16 Statistical analysis

All data were analyzed using two-way analysis of variance (ANOVA), followed by least significant difference (LSD) *post-hoc* tests to assess pairwise differences. Data are presented as mean ± standard deviation (SD). Statistical significance was defined as a *p* < 0.05. All statistical analyses were performed using GraphPad Prism 9.5 software (GraphPad Software, San Diego, CA, United States).

## 3 Result

### 3.1 Network pharmacology

#### 3.1.1 Identification of active components of DEX and prediction of targets for DEX and CIRI

**Figure 1:**
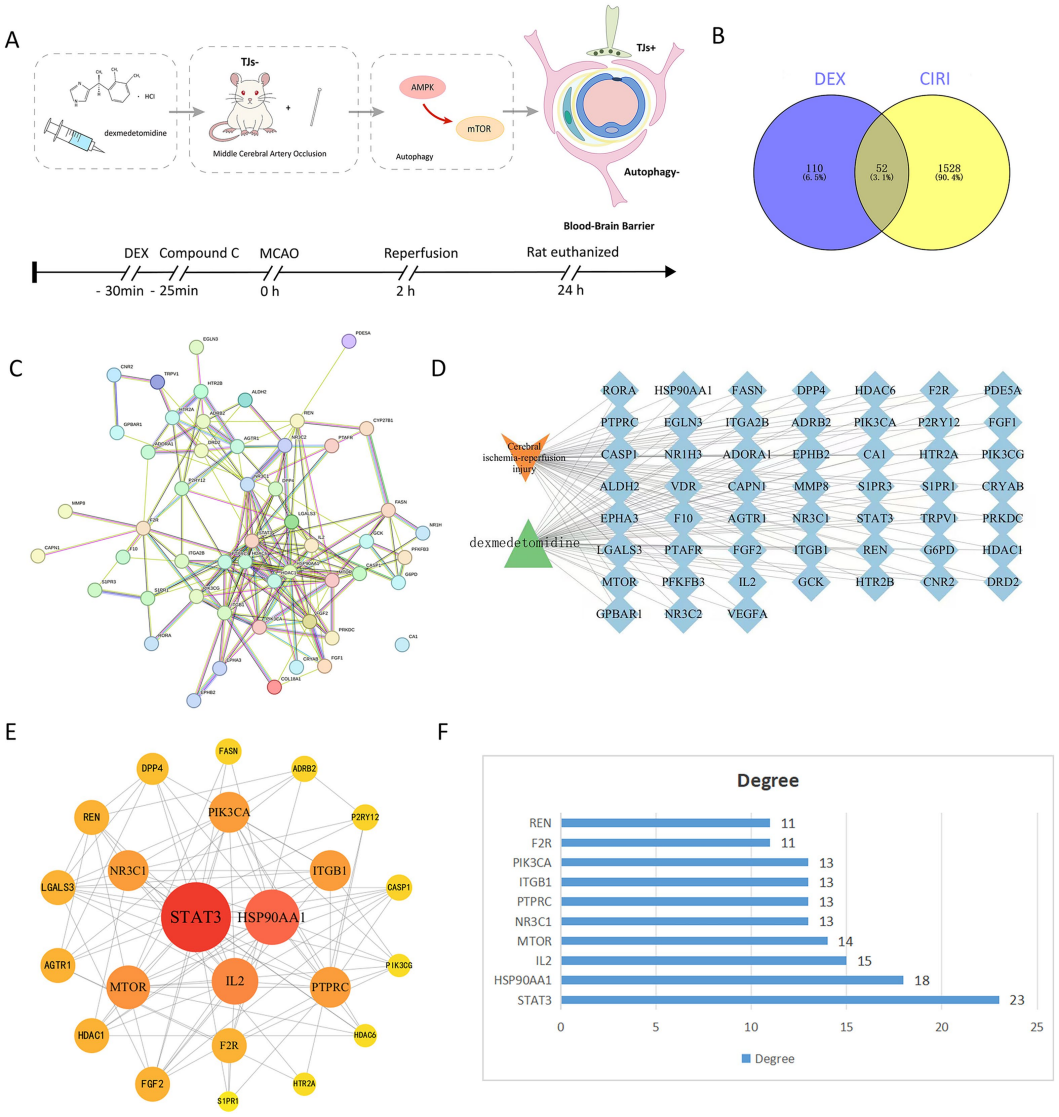
Experimental procedures and network pharmacology-based strategy for predicting the possible mechanisms in DEX for treating CIRI. **(A)** Diagrammatic representation of the experimental procedure. **(B)** The Venn diagram shows the targets in both DEX and CIRI. **(C)** “DEX-target-CIRI” network analysis. **(D)** Topological analysis of the STRING network was performed using 42 commonly used indicators. **(E)** The PPI network. **(F)** Degree plot of the top 10 core targets.

The detailed experimental protocol is illustrated in Figure 1A. Rats were pretreated with DEX via intraperitoneal injection 30 min before model establishment, and received Compound C via intraperitoneal injection 25 min before ischemia induction. Following 2 h of ischemia, reperfusion was performed. Twenty-four hours later, rat brain tissues were collected for subsequent assays. A total of 110 active components were identified using the PubChem database and literature mining. In addition, 1,528 target genes associated with cerebral ischemia reperfusion injury were collected. Venny software was used to identify 52 common targets, which were regarded as potential key targets for DEX in the treatment of cerebral ischemia reperfusion injury (Figure 1B).

#### 3.1.2 “DEX-target-CIRI” network analysis and PPI network

The common targets were imported into Cytoscape 3.9.2 software to construct the “DEX-target-cerebral ischemia reperfusion injury” network diagram. This diagram shows that DEX-related active components may exert therapeutic effects on cerebral ischemia reperfusion injury through these 52 targets (Figure 1C). Subsequently, the PPI network was analyzed using the STRING database, with the species restricted to *Homo sapiens* and the interaction score set to 0.90. Discrete protein nodes were removed to obtain the final PPI network (Figure 1D). Among all nodes, the top 10 key targets were STAT3, HSP90AA1, IL2, mTOR, NR3C1, PIK3CA, ITGB1, PTPRC, F2R, and FGF2 (Figures 1E,F). Therefore, these targets are predicted to be critical for DEX in the treatment of cerebral ischemia reperfusion injury.

#### 3.1.3 GO enrichment analysis and KEGG enrichment analysis

**Figure 2:**
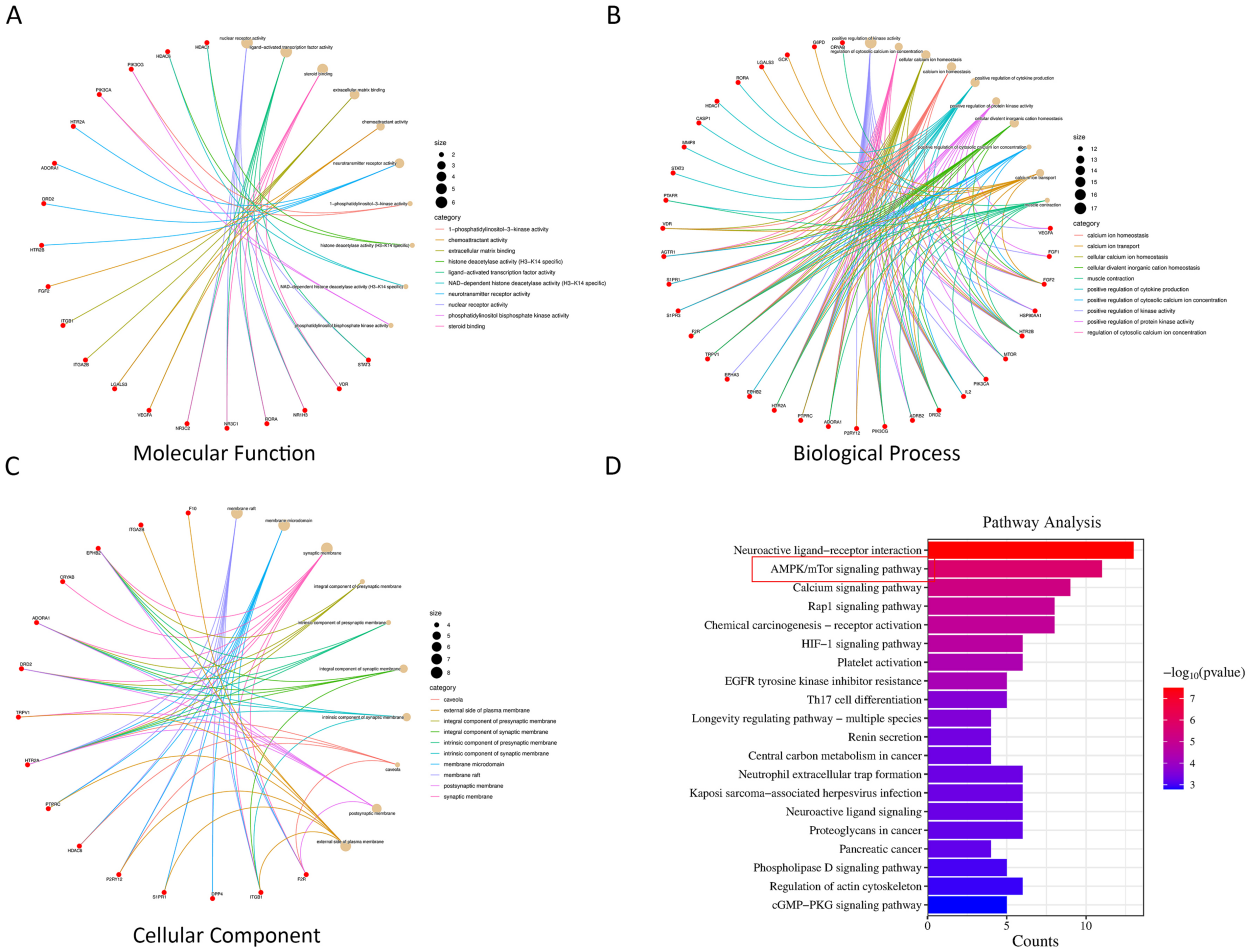
Network pharmacology-driven identification of molecular mechanisms for DEX in mitigating CIRI. **(A–C)** GO enrichment analysis. **(D)** KEGG enrichment analysis.

GO enrichment and KEGG pathway analyses were performed for the targets in the PPI network using the bioinformatics tools ClusterProfiler and STRING software. The top 10 terms with the lowest *p*-values for biological processes (BP), molecular functions (MF), and cellular components (CC) are presented in Figures 2A–C. The key terms in BP included response to lipopolysaccharide, reactive oxygen species metabolic process, and response to reactive oxygen species. The important terms in CC comprised membrane raft, membrane microdomain, and membrane region. The key terms in MF included DNA binding, transcription factor binding, and nuclear receptor activity.

In addition, a total of 257 KEGG pathways were identified by KEGG pathway analysis. In the KEGG enrichment analysis, the count value represents the number of differentially expressed genes annotated to the corresponding pathway. A higher count indicates that more differentially expressed genes are enriched in the pathway, implying a higher likelihood and importance of the pathway being involved in the biological process investigated. As shown in Figure 2D, both the top-ranked pathways and the AMPK pathway exhibited relatively high count values, supporting their potential roles in the protective effects observed in this study. The top 10 KEGG pathways with significantly adjusted *p*-values are displayed in Figure 2D, among which the AMPK/mTOR signaling pathway was identified as a key pathway.

### 3.2 DEX ameliorates neurological deficits in rats with CIRI

**Figure 3:**
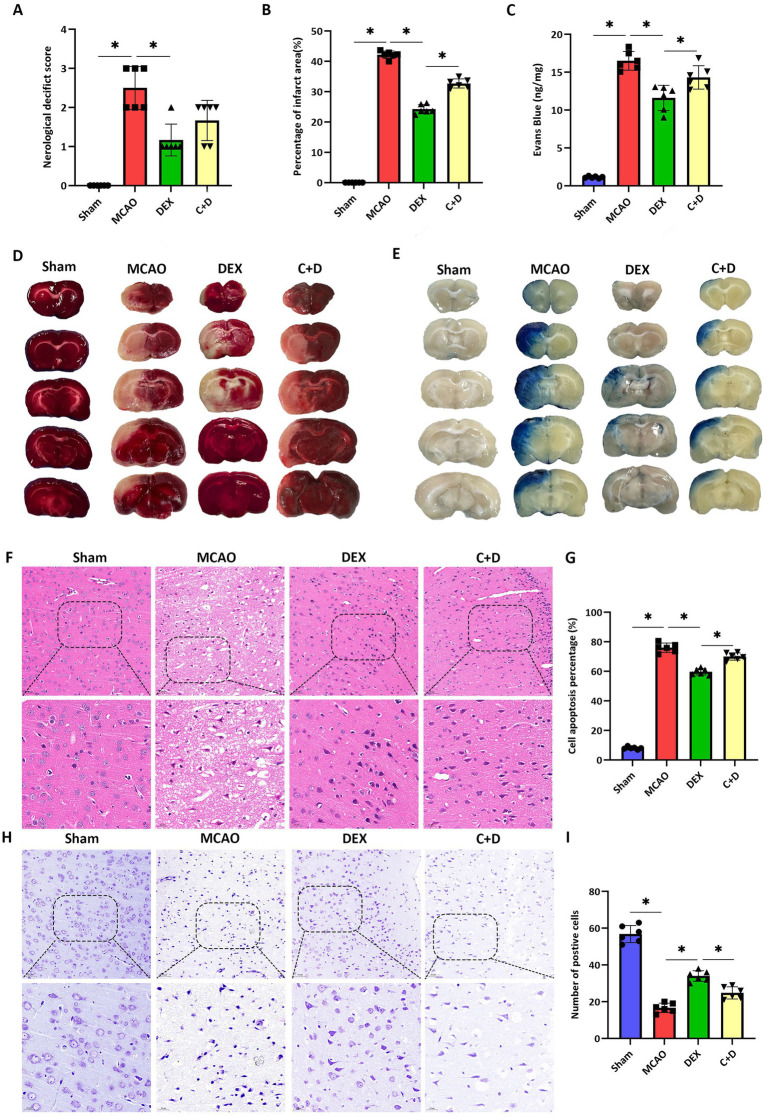
Effects of DEX on nerve and brain injury in rats of each group. **(A)** Neurological function score (*n* = 6). **(B,D)** TTC staining results (*n* = 6). **(C,E)** Evans blue staining results (*n* = 6). **(F,G)** HE staining results (*n* = 6). **(H,I)** Nissl staining results (*n* = 6). Data are expressed as mean ± SEM. ^*^p < 0.05.

The neurological function of rats in each group was evaluated using the Zea-Longa neurological function scoring system. As shown in the results, compared with the Sham group, the neurological function score of rats in the MCAO group was significantly increased (*p* < 0.05). In contrast, the neurological function score of rats in the DEX group was significantly decreased compared with that in the MCAO group (*p* < 0.05). However, there was no statistically significant difference in neurological function scores between the C + D group and the DEX group (*p* < 0.05) (Figure 3A).

### 3.3 DEX pretreatment alleviates brain tissue damage in rats with CIRI

Cerebral infarction in rats was assessed using TTC staining. Compared with the Sham group, the percentage of infarct area was significantly elevated in the MCAO group (*p* < 0.05). Treatment with DEX markedly reduced the infarct area relative to the MCAO group (*p* < 0.05), whereas the infarct area was significantly enlarged in the C + D group compared with the DEX group (*p* < 0.05) (Figures 3B,D). Evans blue staining was further performed to evaluate BBB permeability. Compared with the Sham group, Evans blue extravasation was significantly increased in the MCAO group (*p* < 0.05). DEX treatment notably decreased dye extravasation compared with the MCAO group (*p* < 0.05), while C + D group administration significantly reversed this effect (*p* < 0.05) (Figures 3C,E).

HE staining was performed to evaluate the degree of brain tissue injury in rats from each group. The results of HE staining indicated that compared with the Sham group, the cell apoptosis percentage was significantly increased in the MCAO group (*p* < 0.05). In contrast, the cell apoptosis percentage was significantly decreased in the DEX group compared with the MCAO group (*p* < 0.05); whereas, the cell apoptosis percentage in the C + D group was significantly increased relative to the DEX group (*p* < 0.05) (Figures 3F,G). These results suggest that DEX may exert a protective effect against cerebral injury induced by CIRI.

Nissl staining was employed to assess the survival, morphological integrity of neurons, and the extent of neuronal damage in brain tissue. The results of Nissl staining showed that compared with the Sham group, the number of positive cells was significantly reduced in the MCAO group (*p* < 0.05). In contrast, the number of positive cells in the DEX group was significantly increased compared with that in the MCAO group (*p* < 0.05); while the number of positive cells in the C + D group was significantly decreased compared with the DEX group (*p* < 0.05) (Figures 3H,I).

### 3.4 DEX alleviates BBB structural damage

**Figure 4:**
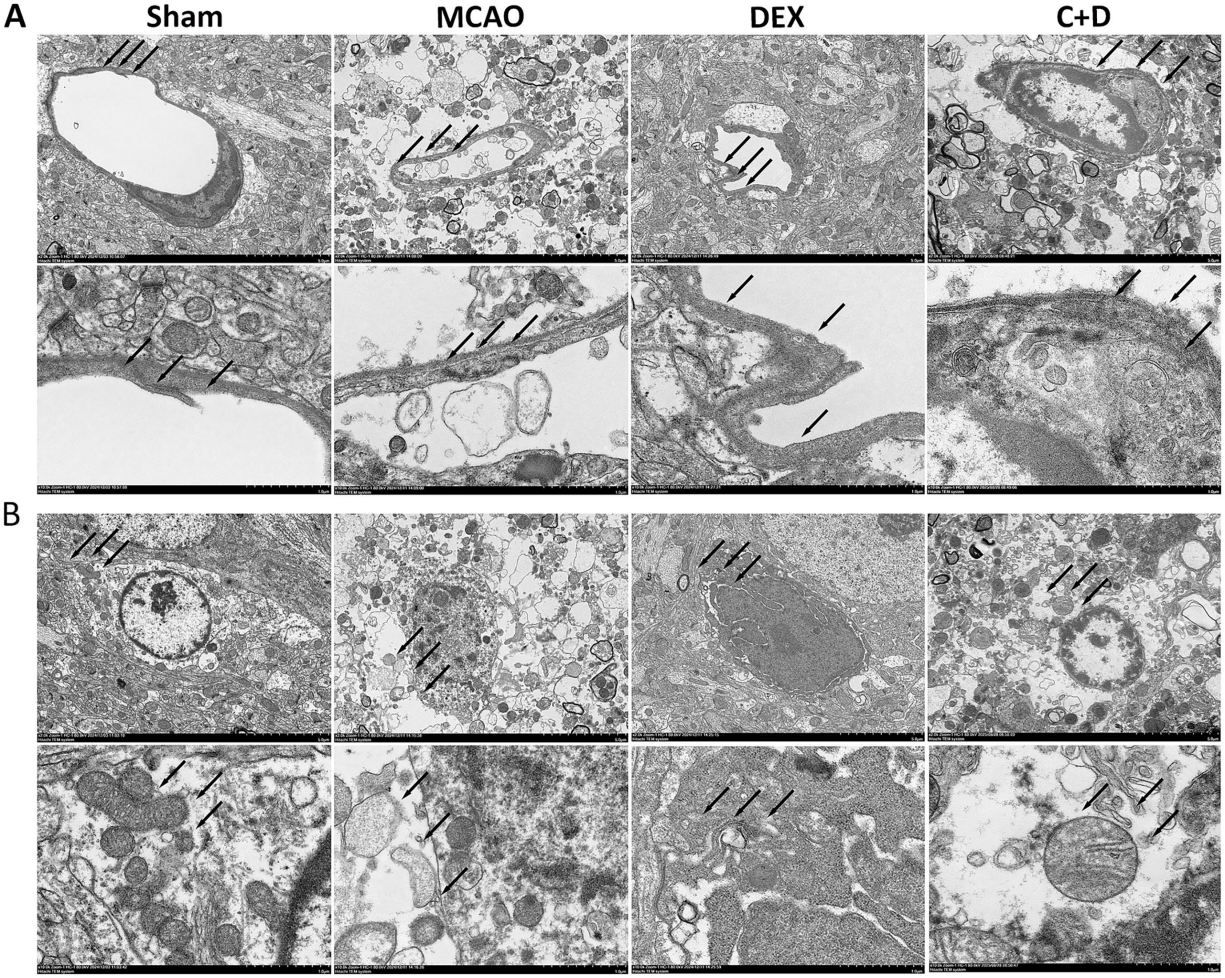
Ultrastructural observation of brain tissue in rats of each group by TEM. **(A)** Structures of TJs (*n* = 3). **(B)** Ultrastructure of autophagosomes (*n* = 3).

Compared with the Sham group, rats in the MCAO group showed disrupted TJs between vascular endothelial cells, severe brain tissue injury, and impaired autophagosome structures. In contrast, compared with the MCAO group, the DEX group exhibited alleviated disruption of tight junctions in vascular endothelial cells, reduced brain tissue damage, and relatively intact autophagosome structures. However, compared with the DEX group, the C + D group still displayed partial disruption of TJs between vascular endothelial cells, impaired autophagosome structures, and localized brain tissue injury (Figures 4A,B).

### 3.5 DEX alleviates motor dysfunction in MCAO rats

**Figure 5:**
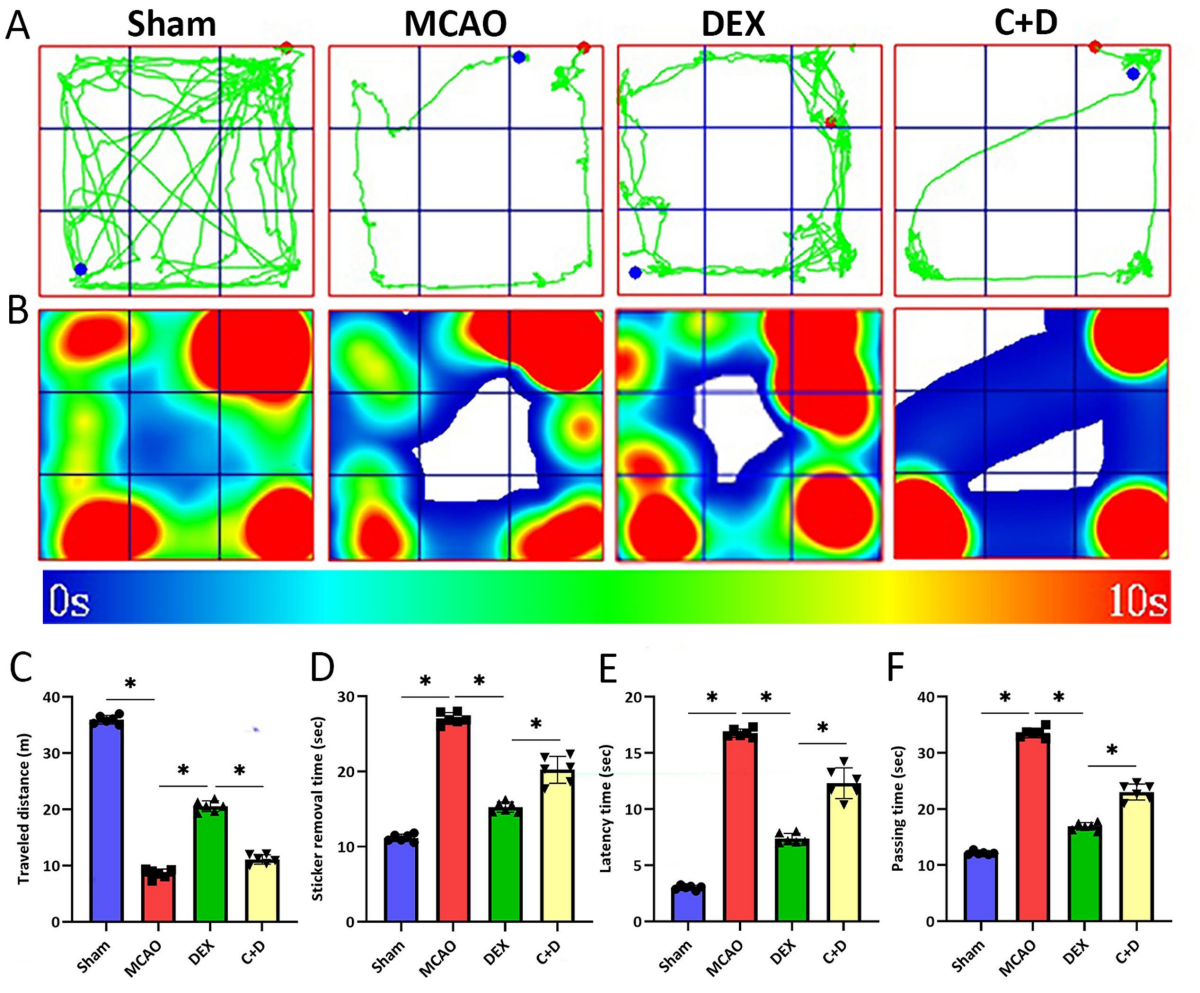
The effect of DEX on the motor ability of rats in each group. **(A)** Open field trajectory map. **(B)** Open field trajectory heatmap. **(C)** Open field traveled distance (*n* = 6). **(D)** Time to remove stickers (*n* = 6). **(E)** Time of latency (*n* = 6). **(F)** Time of passing the balance bar (*n* = 6). Data are expressed as mean ± SEM. ^*^p < 0.05.

Locomotor activity, motor coordination, and somatosensory-motor function in rats were evaluated using the open field test, balance bar test, and sticker removal test, respectively. As shown in Figures 5A–F, open field test results demonstrated that compared with the Sham group, the traveled distance by rats in the MCAO group was significantly shortened (*p* < 0.05), accompanied by a significant prolongation of time to removal stickers, time of latency and passing the balance bar. In contrast, compared with the MCAO group, rats in the DEX group exhibited a significantly increased traveled distance (*p* < 0.05), along with a significant reduction in time to removal stickers, time of latency and passing the balance bar (all *p* < 0.05). However, compared with the DEX group, the traveled distance was significantly decreased in the C + D group (*p* < 0.05), while time to removal stickers, time of latency and passing the balance bar were all significantly prolonged (all *p* < 0.05).

### 3.6 DEX upregulate the expression level of TJs in the brain tissue of MCAO rats

**Figure 6:**
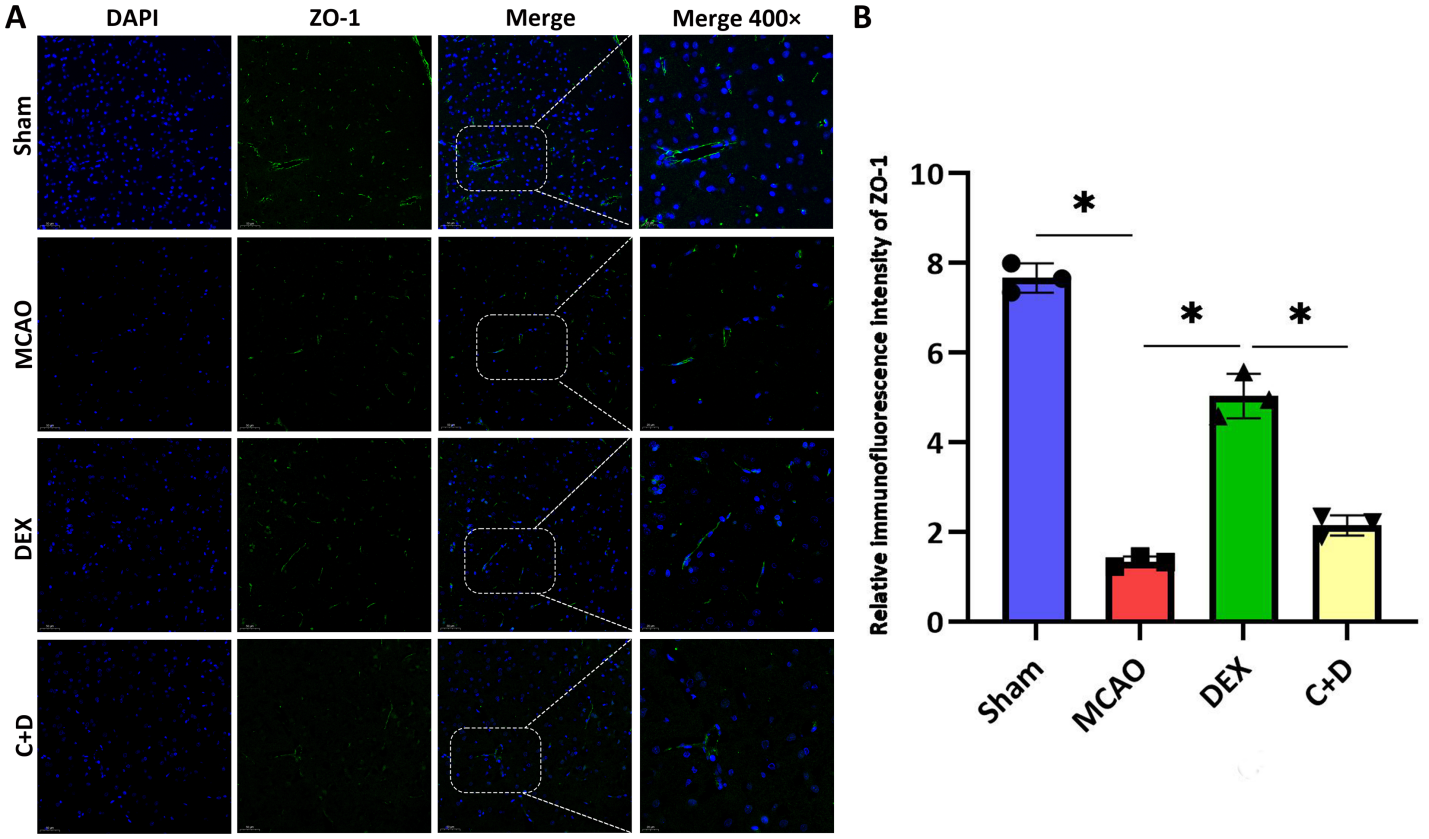
The effect of DEX on the expression level of TJs in rats of each group. **(A,B)** Immunofluorescence expression of ZO-1 (*n* = 3). Data are expressed as mean ± SEM. ^*^p < 0.05.

According to Figures 6A,B, the relative IF density of ZO-1 in brain tissues was significantly decreased in the MCAO group compared with the Sham group (*p* < 0.05). In contrast, the relative IF density of ZO-1was significantly elevated in the DEX group relative to the MCAO group (*p* < 0.05). However, the relative IF density of ZO-1 was markedly reduced in the C + D group compared with the DEX group (*p* < 0.05).

### 3.7 DEX alleviate BBB damage in rats with CIRI via the AMPK/mTOR signaling pathway

**Figure 7:**
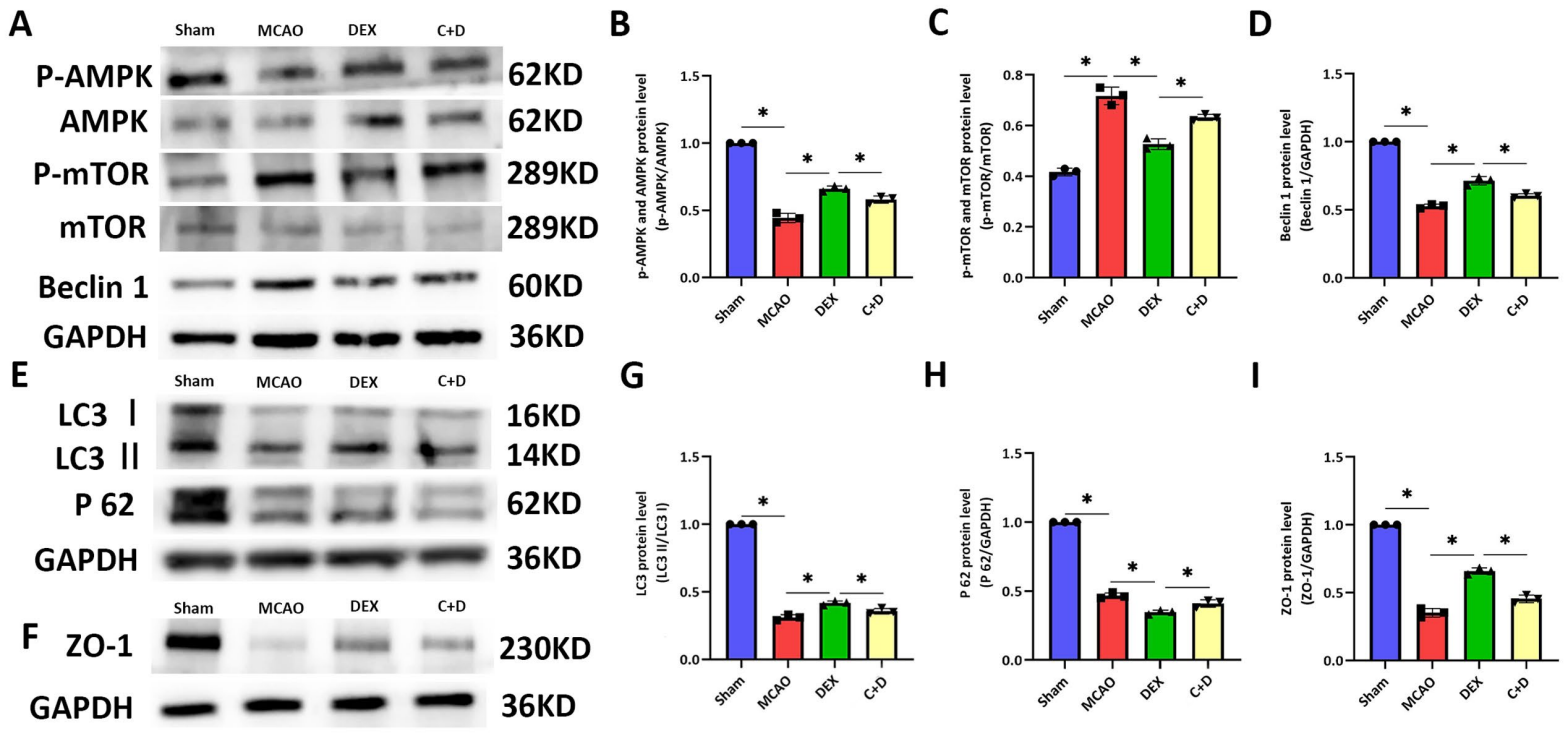
Effects of DEX on the expression levels of related proteins in rats of each group. **(A–D)** Western blot and quantitative analysis of protein levels of p-AMPK/AMPK, p-mTOR/mTOR, and beclin 1 (*n* = 3). **(E–I)** Western blot and quantitative analysis of protein levels of LC3 II/LC3 I, P62, and ZO-1 (*n* = 3). Data are expressed as mean ± SEM. ^*^p < 0.05.

**Figure 8:**
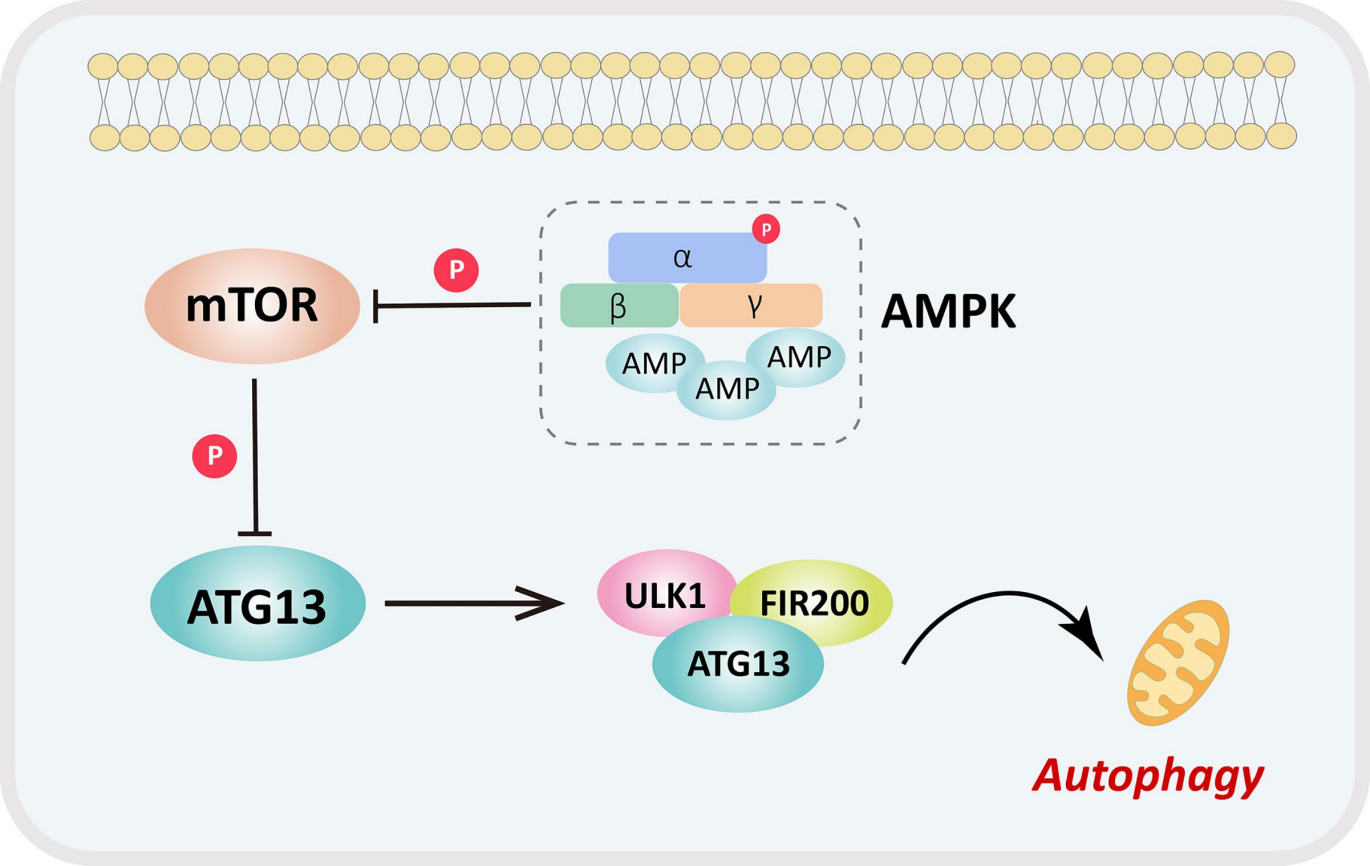
Specific mechanism by DEX improves the BBB and alleviates CIRI induced brain injury via the AMPK/mTOR signal pathway.

As shown in the Western blot results in Figures 7A–I, the ratio of P-AMPK/AMPK, beclin 1, LC3 II/I, and ZO-1 were significantly decreased in the MCAO group compared with the Sham group (all *p* < 0.05), while the ratio of P-mTOR/mTOR and P62 were significantly increased (all *p* < 0.05). In contrast, the ratio of P-AMPK/AMPK, beclin 1, LC3 II/I, and ZO-1 were significantly elevated in the DEX group compared with the MCAO group (all *p* < 0.05), whereas the ratio of P-mTOR/mTOR and P62 were significantly reduced (all *p* < 0.05). Furthermore, compared with the DEX group, the ratio of P-AMPK/AMPK, beclin 1, LC3 II/I, and ZO-1 were significantly decreased in the C + D group (all *p* < 0.05), while the ratio of P-mTOR/mTOR and P62 were significantly increased (all *p* < 0.05). These results indicate that DEX alleviate the structural damage of the BBB by activating the AMPK/mTOR signaling pathway, thereby improving the pathological condition of CIRI (see Figure 8).

## 4 Discussion

Structural and functional impairment of the BBB represents a pivotal event in the pathophysiological cascade of ischemic stroke, which aggravates secondary neuronal damage and contributes to unfavorable clinical outcomes (Shi et al., 2025). Therefore, exploring effective interventions to alleviate BBB disruption following CIRI has become an urgent clinical concern. Although previous studies have demonstrated that DEX alleviates CIRI induced inflammation to a certain degree, the precise molecular mechanisms by which DEX mediated BBB protection occurs following CIRI remain largely unknown (Zhao et al., 2021). Accordingly, this study aimed to systematically explore the effects of DEX on BBB integrity in a rat model of CIRI.

In this study, 52 key indicators related to DEX and CIRI were obtained using network topology methods. KEGG and GO enrichment analyses revealed that DEX may alleviate CIRI through autophagy related signaling pathways. Furthermore, KEGG analysis indicated that DEX treatment can significantly reduce brain damage, among which the AMPK/mTOR signaling pathway may play a crucial role. Meanwhile, relevant experiments conducted on CIRI model rats showed that DEX pretreatment improved the structural damage of the BBB in CIRI rats, thereby further alleviating the condition.

The MCAO model is a well-recognized animal model widely used to investigate CIRI induced brain damage and its potential therapeutic mechanisms (Longa et al., 1989). Specifically, focal cerebral ischemia was induced by inserting a monofilament suture to occlude the middle cerebral artery. After 2 h of ischemia, reperfusion was established by withdrawing the suture. Tissue samples were harvested 24 h after model establishment to simulate the pathological process of CIRI in clinical practice. Using MCAO rats as research subjects, neurological function scoring, the open field test, the sticker removal test, and the balance bar test were utilized to evaluate the effects of DEX treatment on the behavioral capabilities of the rats. The results demonstrated that DEX pretreatment prolonged the open field traveled distance, reduced the sticker removal time, balance bar latency, and bar passing time, and decreased the neurological function score in MCAO rats. These findings suggest that DEX treatment significantly ameliorates neurological and motor dysfunction in MCAO rats. The pathogenesis of CIRI is mainly attributed to ischemia and hypoxia in brain tissue (Quan et al., 2025). The cerebral cortex, which is directly associated with ischemia and hypoxia, is highly vulnerable to these insults. To further clarify the molecular mechanism by which DEX ameliorates brain injury in MCAO rats, this study focused on the cerebral cortex. Experimental results showed that after DEX pretreatment, HE staining revealed improved structural damage in the cortical region and a reduced rate of cell apoptosis in MCAO rats, which was consistent with previous studies. Nissl staining analysis confirmed a decrease in the number of damaged Nissl bodies following DEX treatment. TTC staining also indicated that DEX could reduce the percentage of infarct area in MCAO rats, suggesting a certain neuroprotective effect. Additionally, Evans blue staining results demonstrated that DEX reduced dye extravasation in the brain tissue of MCAO rats, confirming that DEX exerts a pharmacological effect in alleviating structural damage to the BBB. Current research indicates that DEX exerts a neuroprotective effect on CIRI rats, significantly improving neuronal injury and BBB structural damage in these rats. This provides a potential approach for the prevention and treatment of CIRI.

BBB disruption constitutes a critical pathological feature in CIRI. Structural impairment of the BBB typically leads to increased permeability and impaired regulation of substance transport, resulting in vasogenic brain edema and reduced clearance of intracerebral waste products—factors that further exacerbate pathological progression (Feng Y. A. et al., 2025). Our study demonstrated that in MCAO model rats, the immunofluorescence intensity of ZO-1was significantly reduced, accompanied by a significant decrease in their protein expression levels. Meanwhile, TEM observations revealed that DEX pretreatment attenuated vascular endothelial cell injury in MCAO rats, preserved the structural integrity of TJs, and increased the number of autophagosomes. These results indicate that DEX effectively alleviates BBB damage induced by CIRI.

Autophagy and pyroptosis interact with each other in CIRI, and the AMPK/mTOR signaling pathway is important for regulating the crosstalk between autophagy and pyroptosis (Fu et al., 2025). AMPK acts as a sensor of cellular energy status and plays an important role in maintaining energy homeostasis (Samad et al., 2025). It serves as a positive regulator of autophagy and can modulate energy metabolism by downregulating mTOR phosphorylation (Hoseini et al., 2025). As a cellular energy sensor, AMPK regulates multiple pathological processes, including protein synthesis, autophagy, and cell death (Li et al., 2025). During cellular energy stress, AMPK phosphorylates mTORC1 and other downstream targets, which directly promotes autophagy (Gan et al., 2025). By fusing autophagosomes with lysosomes to form autolysosomes, which degrade toxic substances and inflammatory components such as NLRP3, autophagy generally inhibits pyroptosis. Our results demonstrated that DEX upregulated the protein expression of P-AMPK/AMPK, beclin 1, LC3 II/I, and ZO-1, and downregulated the P-mTOR/mTOR ratio and P62 expression. These findings suggest that DEX alleviates BBB structural damage induced by CIRI by regulating the AMPK/mTOR signaling pathway, providing a novel insight for the clinical treatment of CIRI.

This study has several limitations. First, the AMPK/mTOR signaling pathway involves multiple upstream and downstream regulatory factors, which warrants further investigation. Second, the intricate nature of BBB interactions and the moderate sample size of the studied variables make it impossible to identify many BBB mechanisms. Finally, this study only used animal brain tissue, which has limitations in the localization detection of endothelial cells. Meanwhile, no *in vitro* cellular experiments were performed for further verification. Therefore, the present study is still incomplete.

## 5 Conclusion

In conclusion, our study demonstrates that DEX treatment preserves the structural integrity of the BBB by upregulating the expression of TJs, thereby alleviating CIRI. The activation of the AMPK/mTOR signaling pathway may contribute to the neuroprotective effect of DEX. In addition, targeting the BBB may represent a promising therapeutic strategy for CIRI. Although DEX exhibits potential in ameliorating CIRI pathology, future studies are warranted to further investigate the bioavailability of DEX and its translational therapeutic potential.

## Data Availability

The original contributions presented in the study are publicly available. This data can be found here: https://doi.org/10.5061/dryad.q83bk3jxz.
