# Supplementary figures and images for: Exploring the molecular mechanism of dexmedetomidine in alleviating blood–brain barrier disruption in rats with cerebral ischemia reperfusion injury based on network pharmacology
Source: Front Mol Neurosci. 2026 Apr 8;19:1750882. doi: 10.3389/fnmol.2026.1750882 (PMC13099826; doi:10.3389/fnmol.2026.1750882)

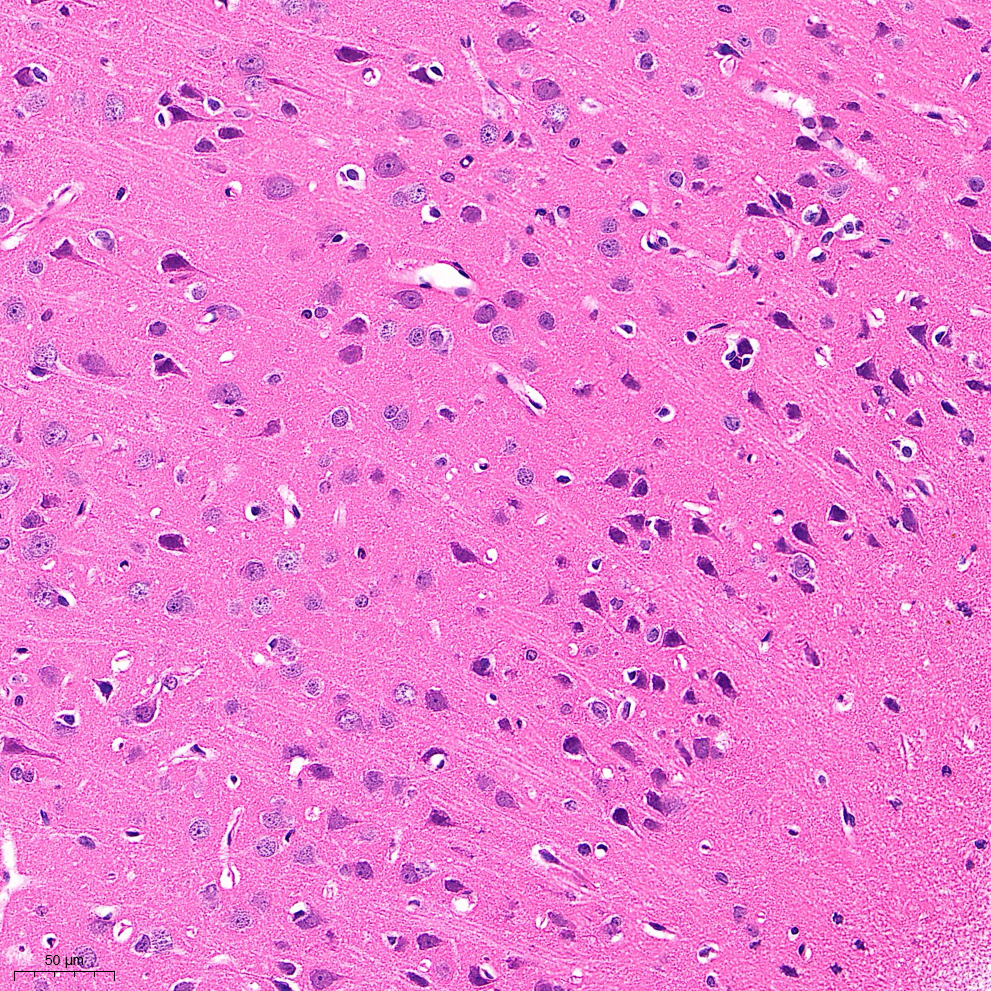

Supplement: Supplementary file 1 [file Data_Sheet_1.ZIP › FIG4/50.svs_20.0x.jpg]

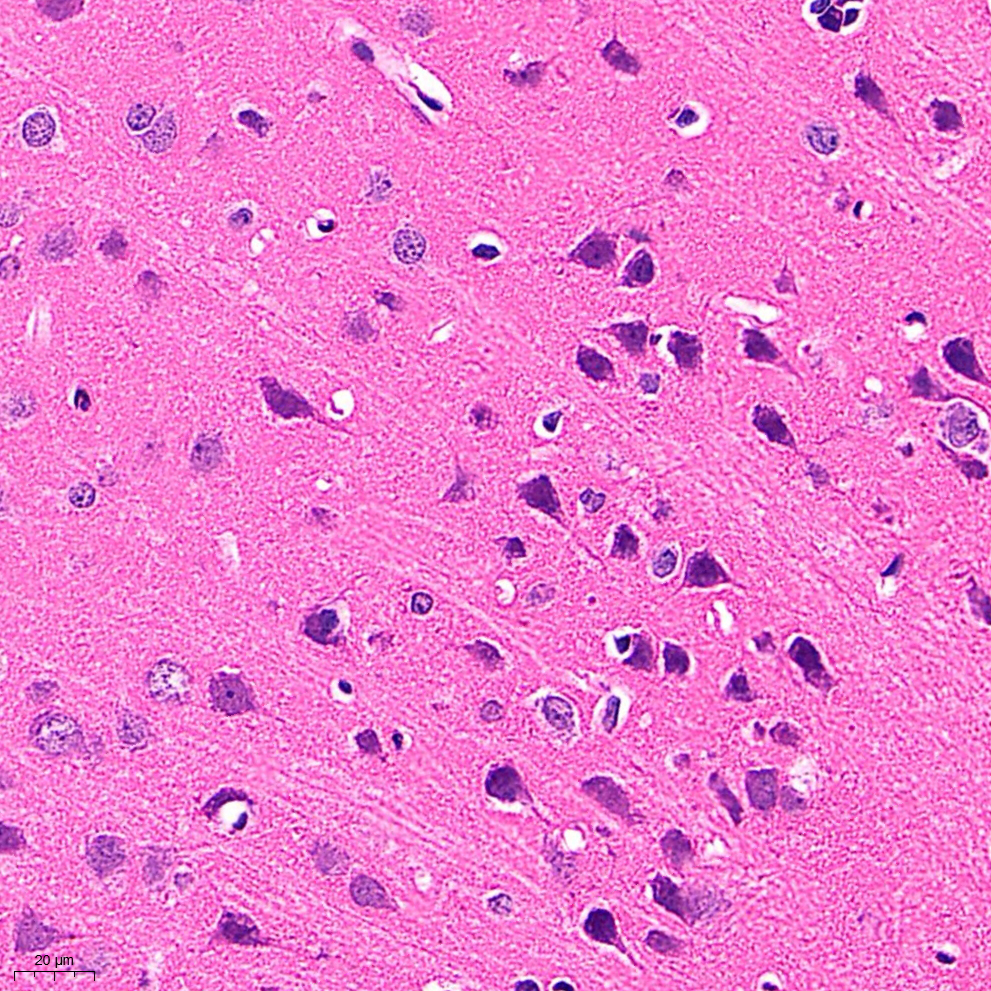

Supplement: Supplementary file 1 [file Data_Sheet_1.ZIP › FIG4/50.svs_40.0x.jpg]

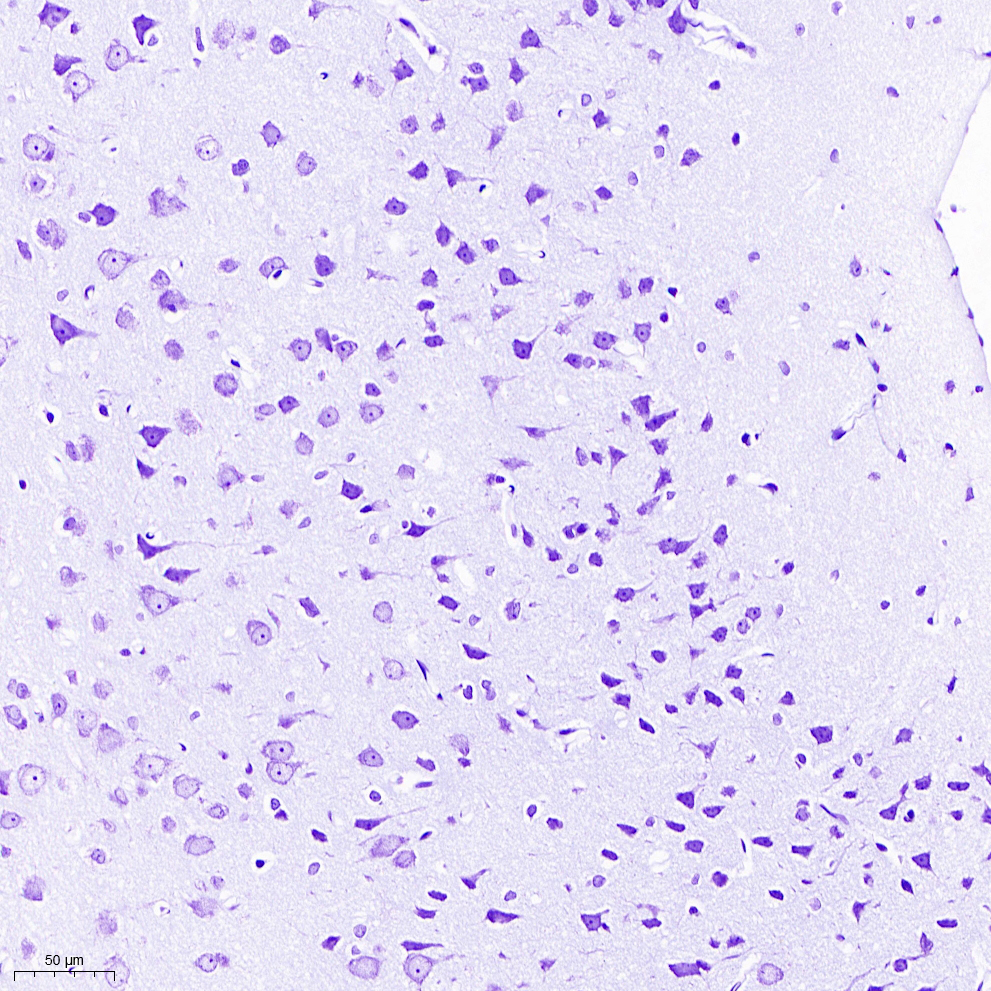

Supplement: Supplementary file 1 [file Data_Sheet_1.ZIP › FIG4/50_20.0x.jpg]

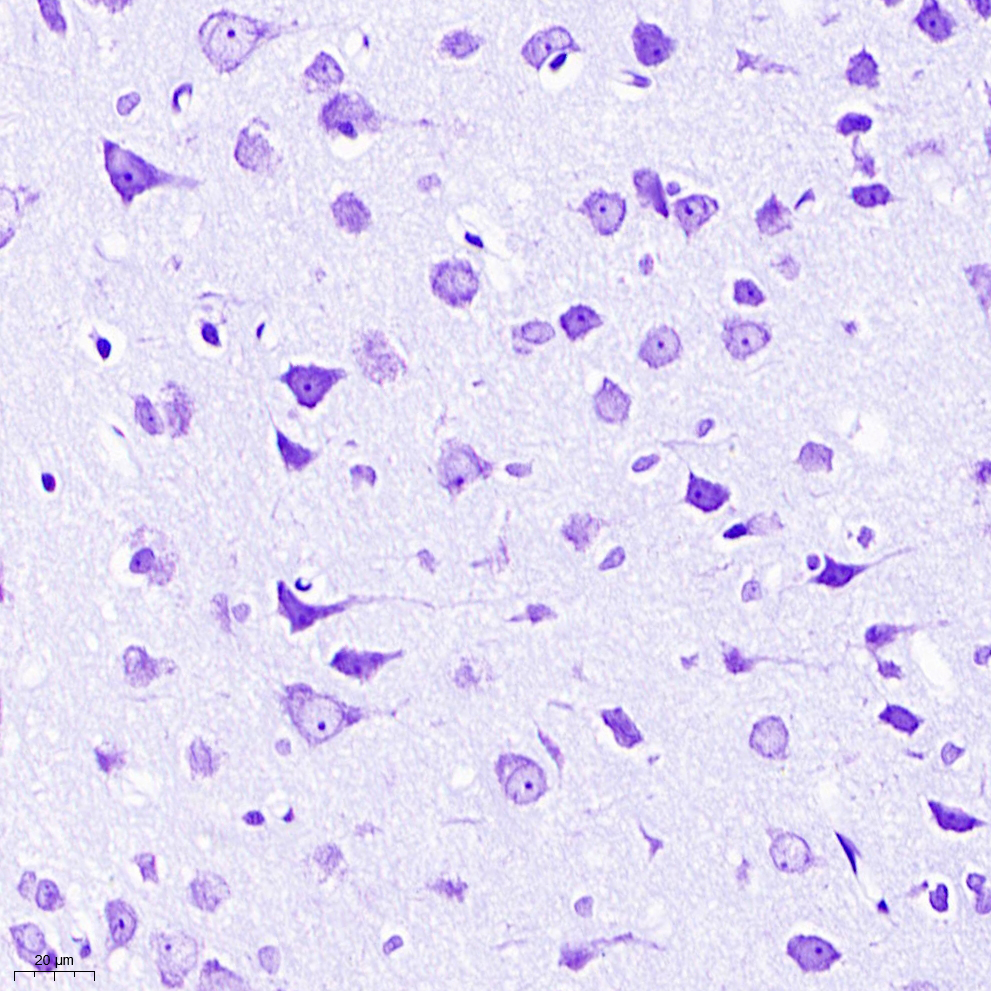

Supplement: Supplementary file 1 [file Data_Sheet_1.ZIP › FIG4/50_40.0x.jpg]

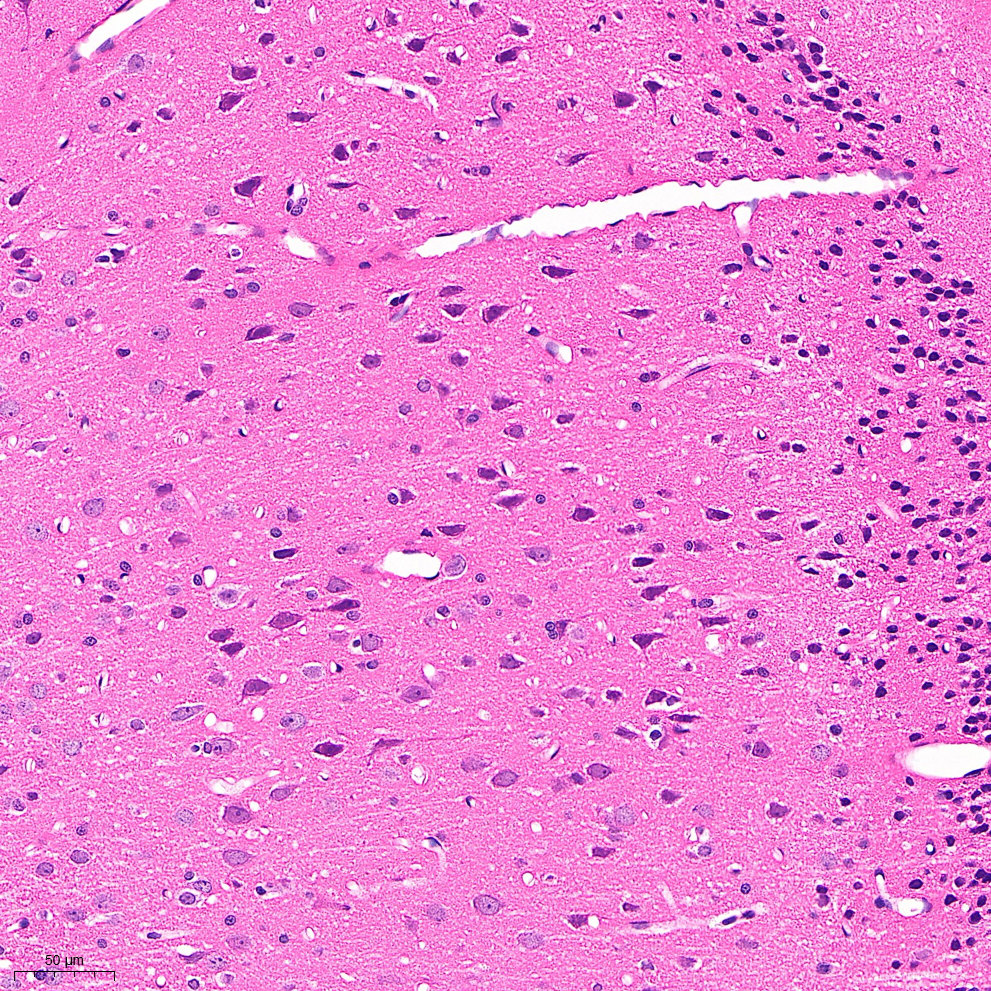

Supplement: Supplementary file 1 [file Data_Sheet_1.ZIP › FIG4/C.svs_20.0x.jpg]

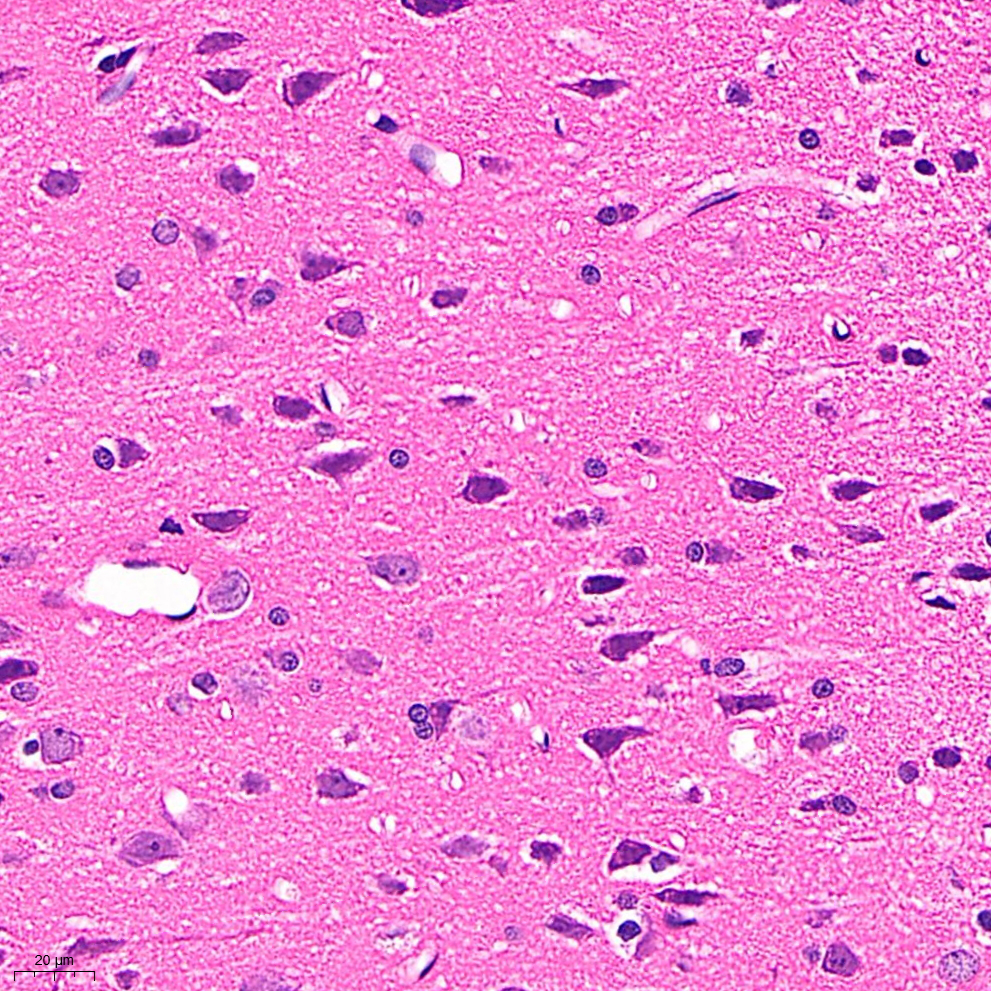

Supplement: Supplementary file 1 [file Data_Sheet_1.ZIP › FIG4/C.svs_40.0x.jpg]

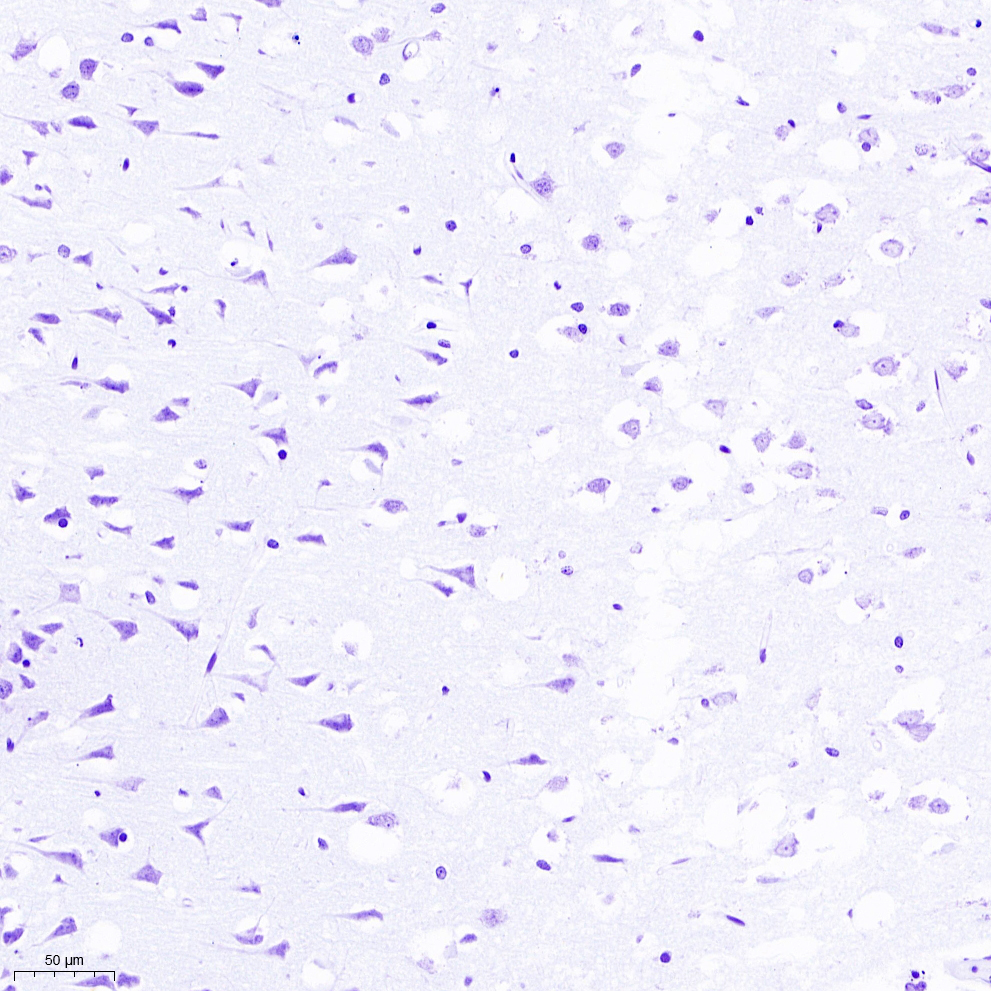

Supplement: Supplementary file 1 [file Data_Sheet_1.ZIP › FIG4/C_20.0x.jpg]

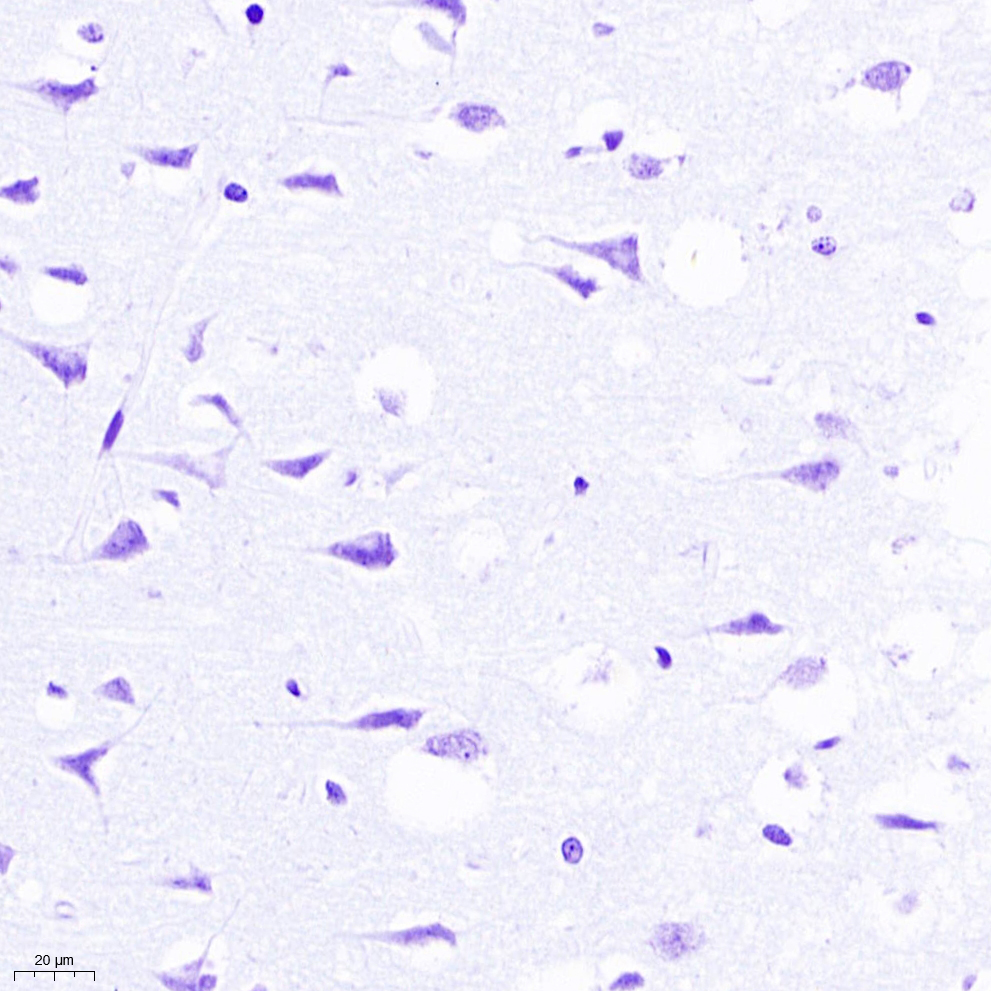

Supplement: Supplementary file 1 [file Data_Sheet_1.ZIP › FIG4/C_40.0x.jpg]

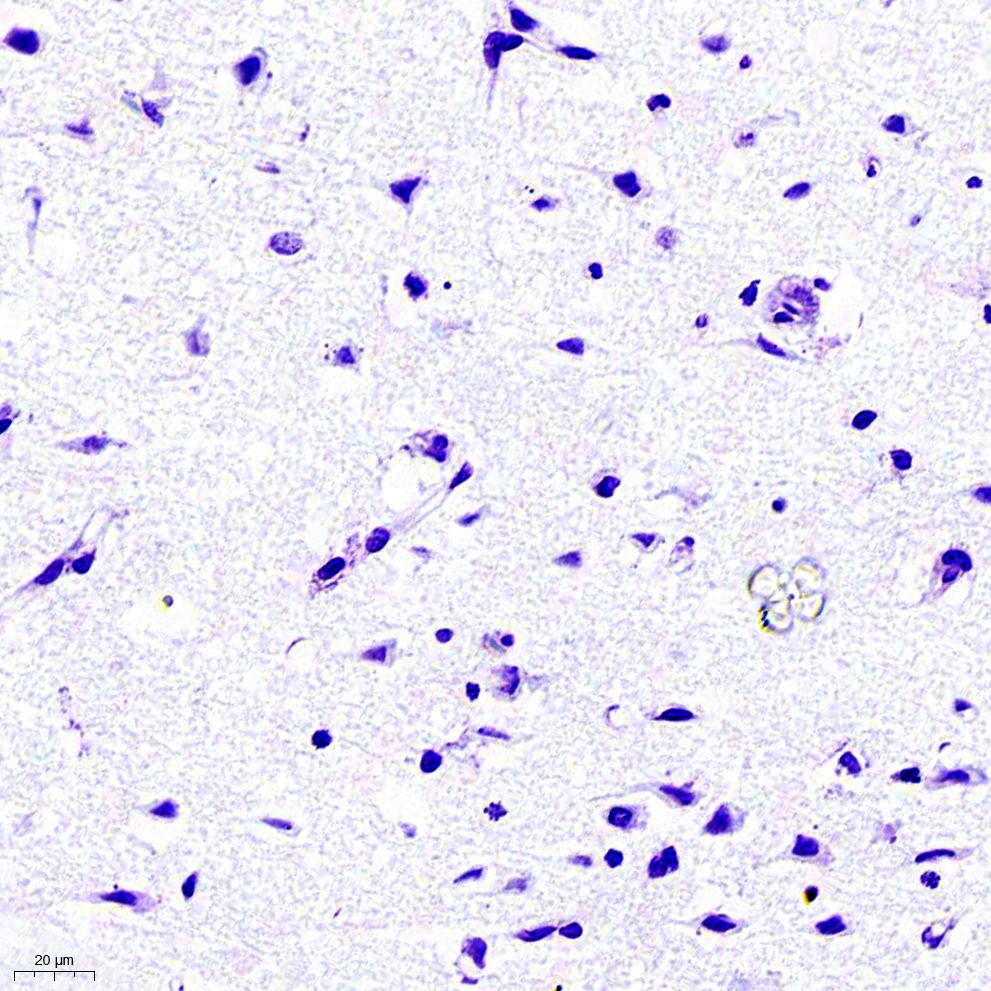

Supplement: Supplementary file 1 [file Data_Sheet_1.ZIP › FIG4/M M40.0x.jpg]

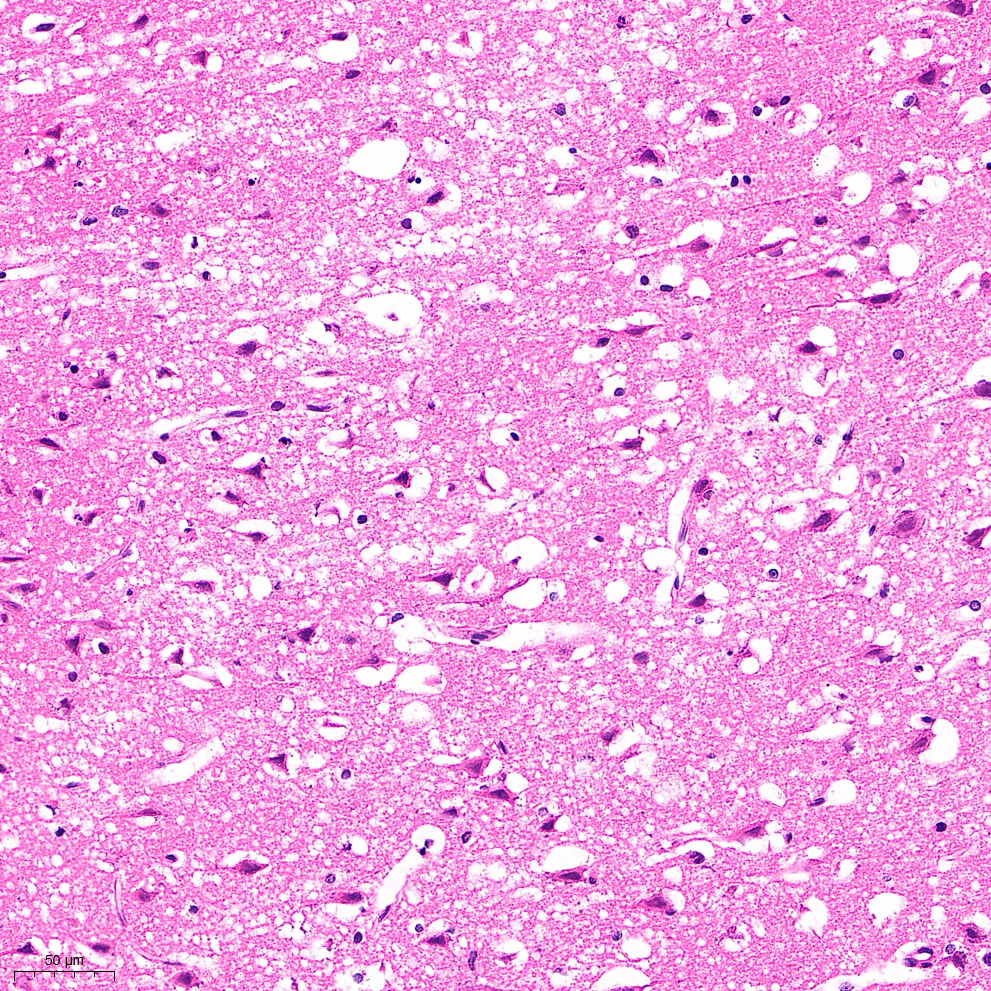

Supplement: Supplementary file 1 [file Data_Sheet_1.ZIP › FIG4/M.svs_20.0x.jpg]

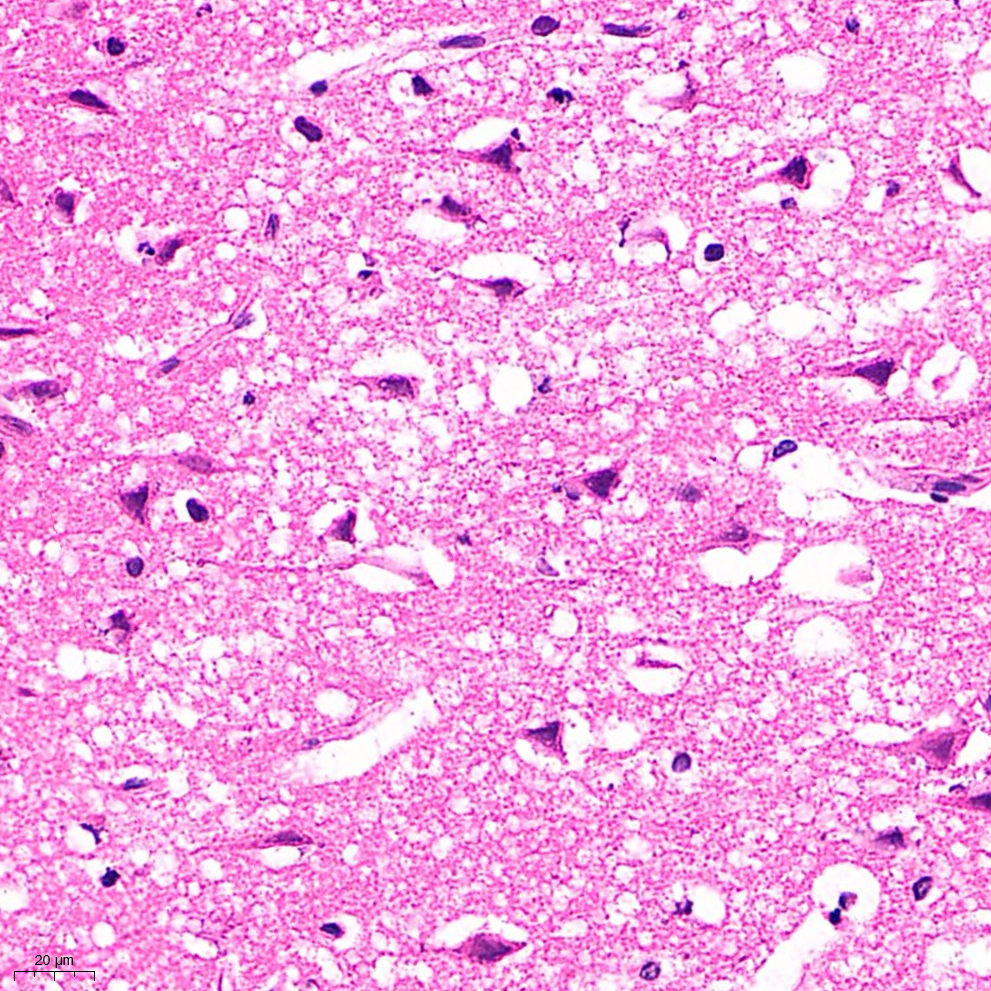

Supplement: Supplementary file 1 [file Data_Sheet_1.ZIP › FIG4/M.svs_40.0x.jpg]

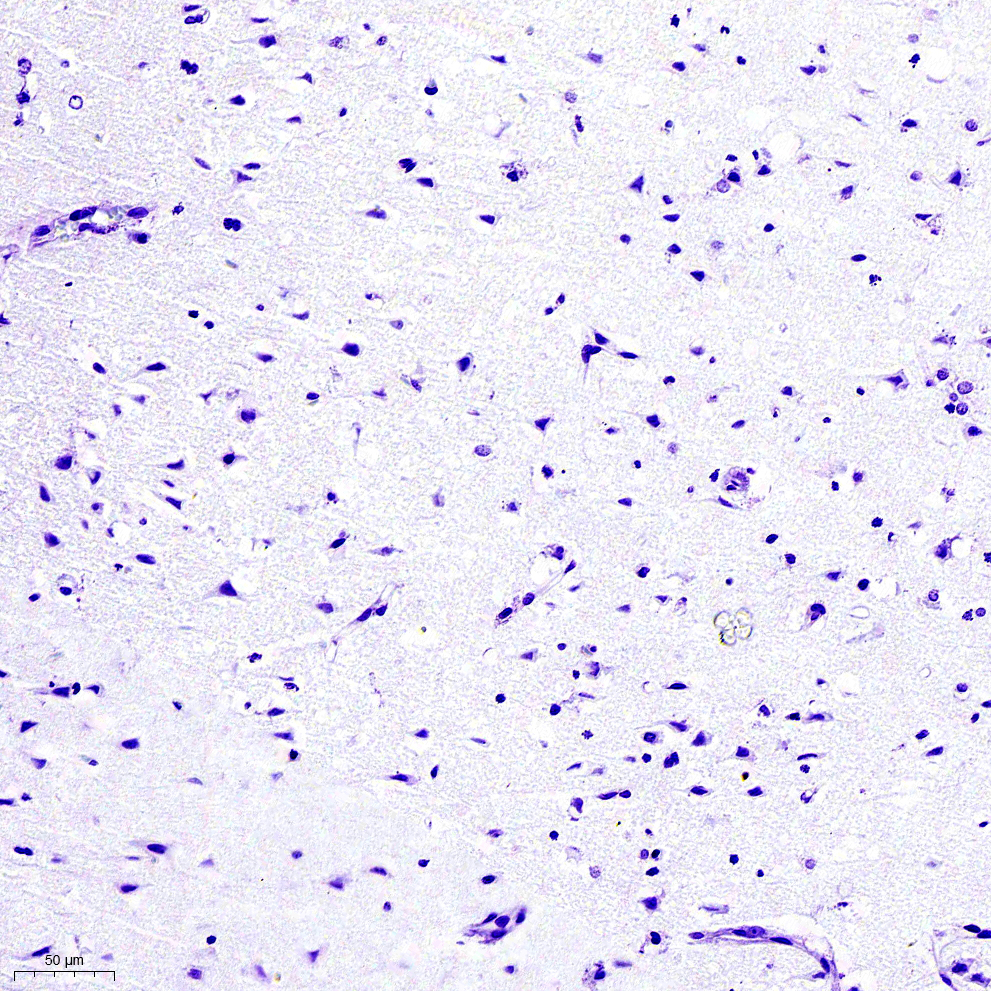

Supplement: Supplementary file 1 [file Data_Sheet_1.ZIP › FIG4/MM _20.0x.jpg]

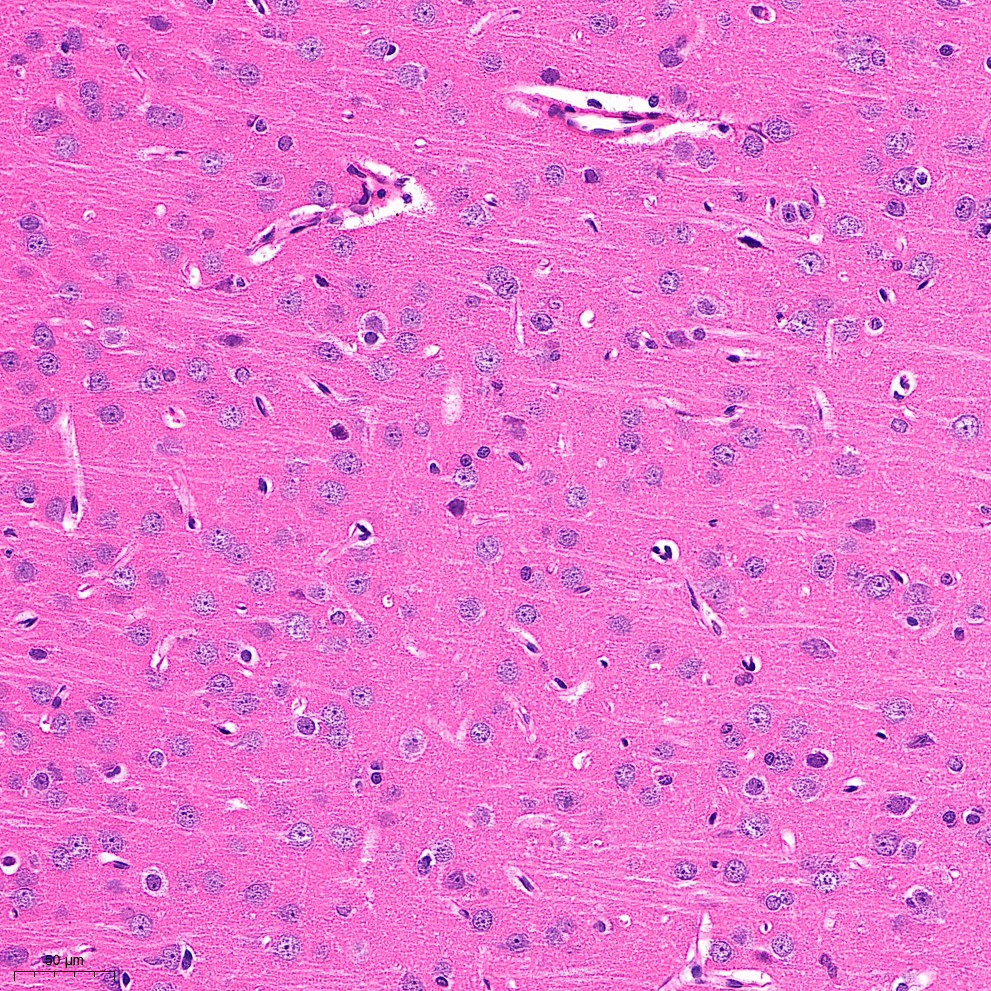

Supplement: Supplementary file 1 [file Data_Sheet_1.ZIP › FIG4/S.svs_20.0x.jpg]

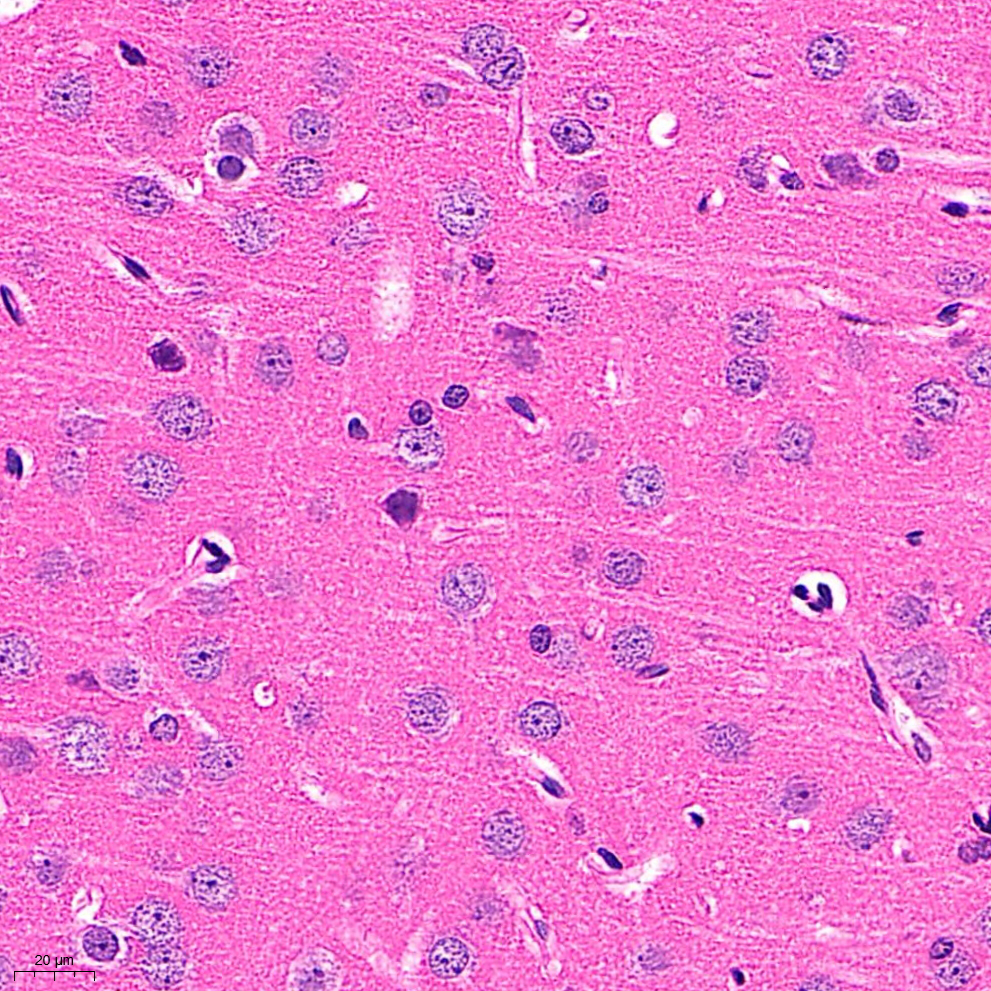

Supplement: Supplementary file 1 [file Data_Sheet_1.ZIP › FIG4/S.svs_40.jpg]

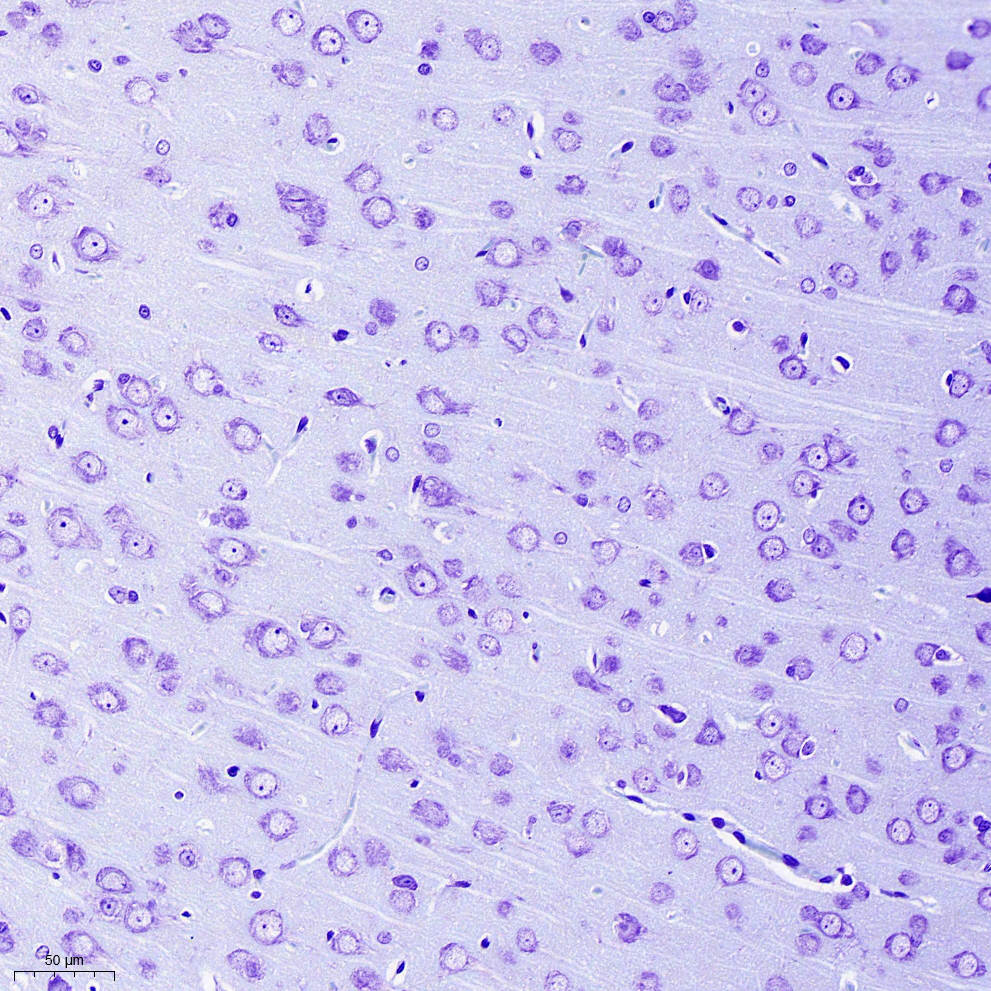

Supplement: Supplementary file 1 [file Data_Sheet_1.ZIP › FIG4/s_20.0x.jpg]

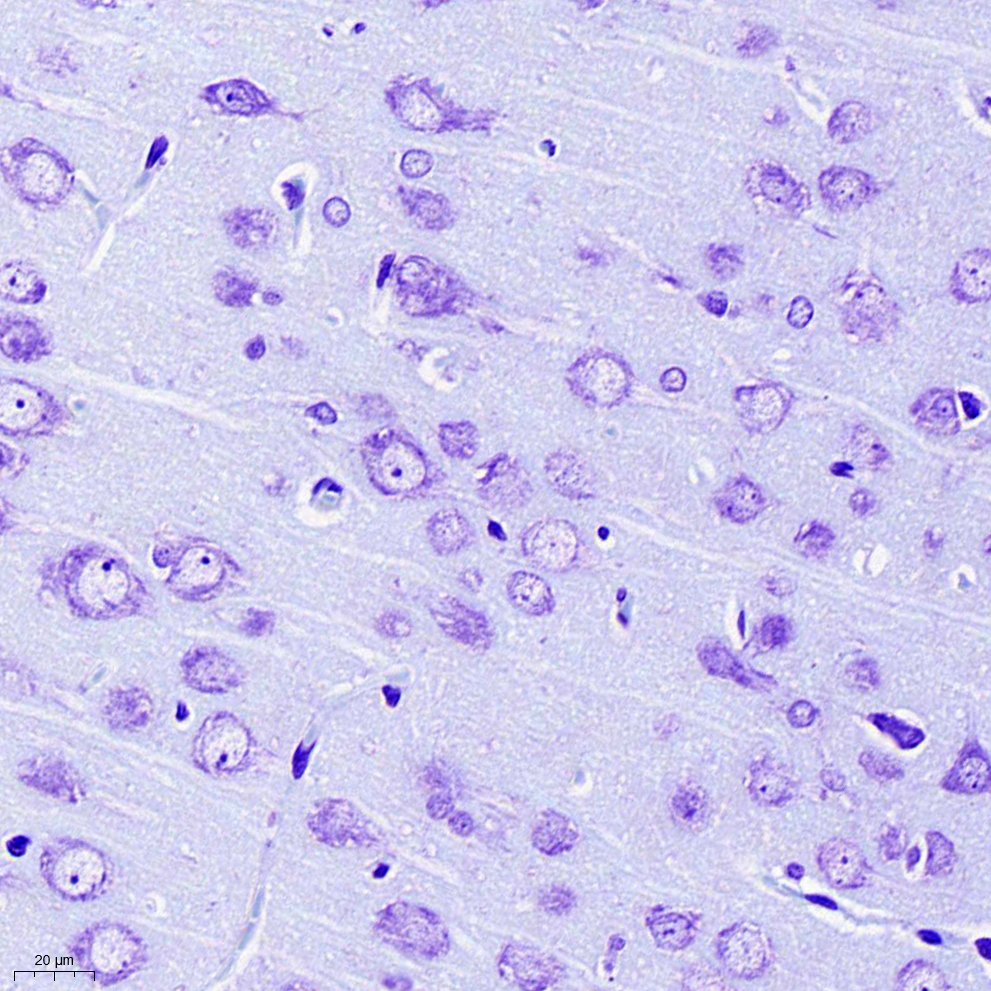

Supplement: Supplementary file 1 [file Data_Sheet_1.ZIP › FIG4/s_40.0x.jpg]

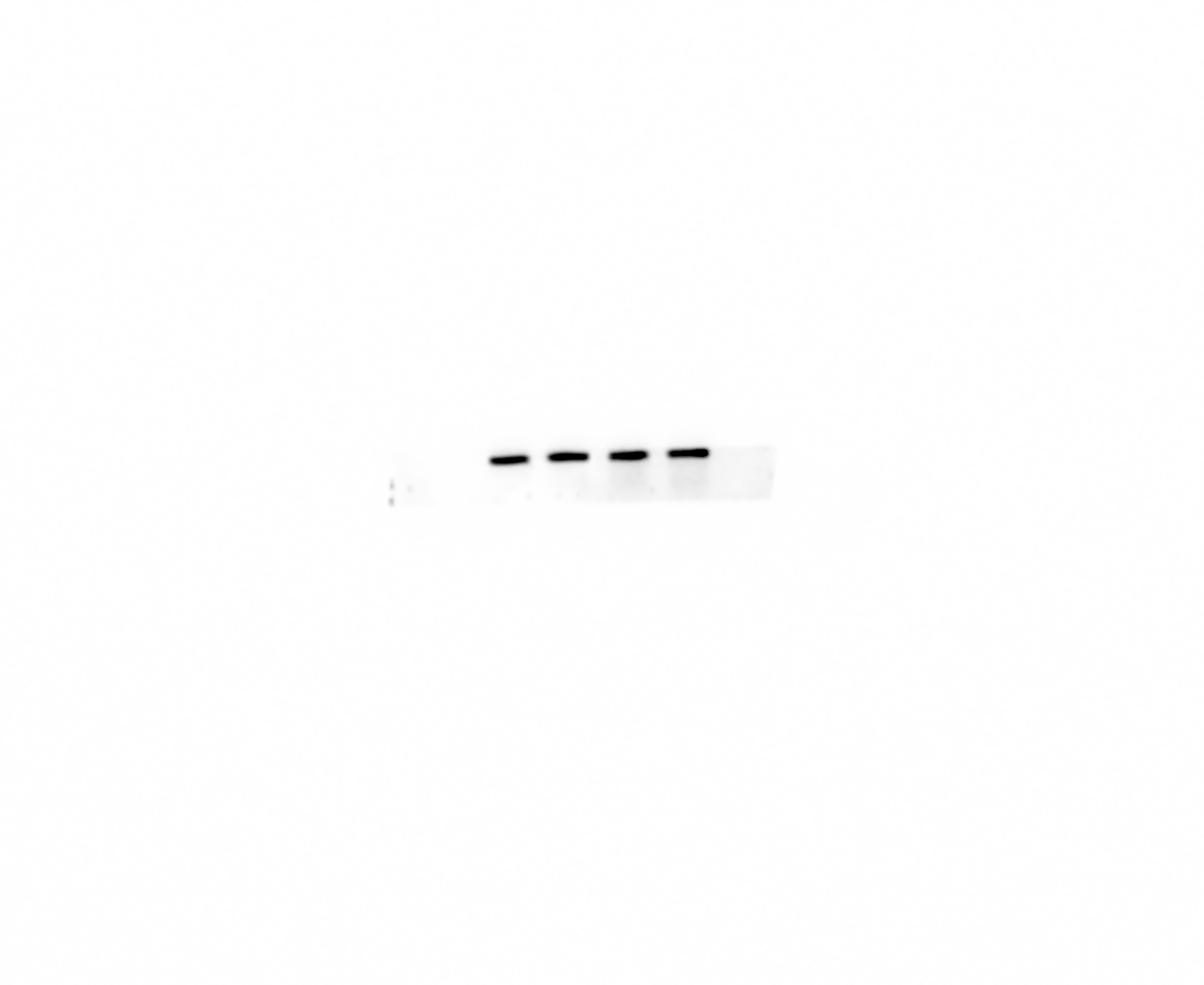

Supplement: Supplementary file 3 [file Image_2.JPEG]

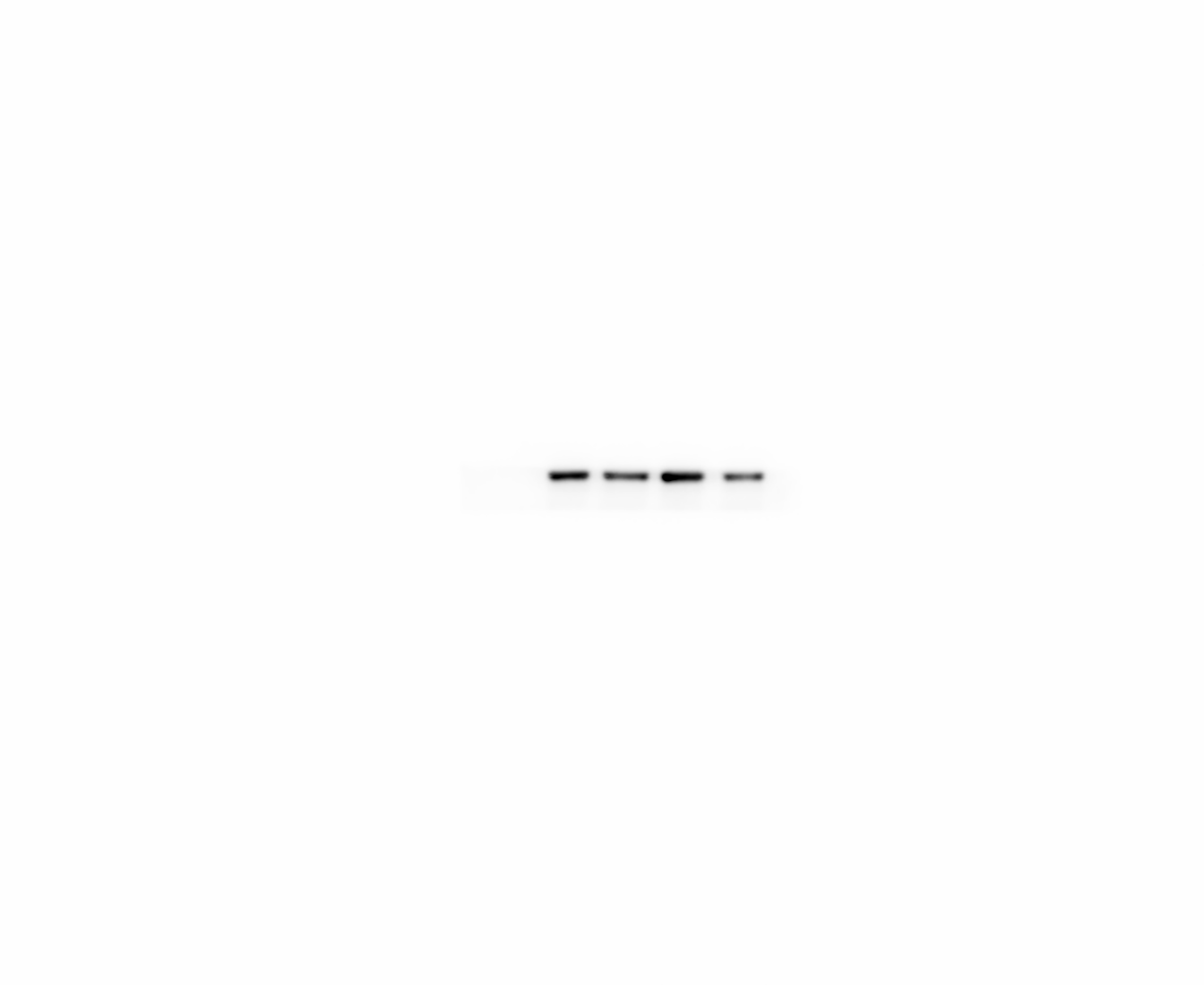

Supplement: Supplementary file 10 [file Image_9.TIF]
